# Imidazole propionate ameliorates atopic dermatitis-like skin lesions by inhibiting mitochondrial ROS and mTORC2

**DOI:** 10.3389/fimmu.2024.1324026

**Published:** 2024-03-12

**Authors:** Ha Eun Kim, Jong Yeong Lee, Dong-Hoon Yoo, Hyo-Hyun Park, Eun-Ju Choi, Kyung-Hwa Nam, Jin Park, Jin Kyeong Choi

**Affiliations:** ^1^ Department of Immunology, Jeonbuk National University Medical School, Jeonju, Republic of Korea; ^2^ Department of Sports Rehabilitation and Exercise Management, University of Gyeongnam Geochang, Geochang-gun, Republic of Korea; ^3^ Department of Clinical Pathology, Daegu Health College, Daegu, Republic of Korea; ^4^ Department of Physical Education, College of Education, Daegu Catholic University, Gyeongsan, Republic of Korea; ^5^ Department of Dermatology, Jeonbuk National University Medical School, Jeonju, Republic of Korea; ^6^ Research Institute of Clinical Medicine of Jeonbuk National University-Biomedical Research Institute of Jeonbuk National University Hospital, Jeonju, Republic of Korea

**Keywords:** imidazole propionate, atopic dermatitis, mitochondria ROS, mTORC2, AMPK, DDIT4

## Abstract

**Background:**

Imidazole propionate (IMP) is a histidine metabolite produced by some gut microorganisms in the human colon. Increased levels of IMP are associated with intestinal inflammation and the development and progression of cardiovascular disease and diabetes. However, the anti-inflammatory activity of IMP has not been investigated. This study aimed to elucidate the role of IMP in treating atopic dermatitis (AD).

**Methods:**

To understand how IMP mediates immunosuppression in AD, IMP was intraperitoneally injected into a *Dermatophagoides farinae* extract (DFE)/1-chloro-2,4 dinitrochlorobenzene (DNCB)-induced AD-like skin lesions mouse model. We also characterized the anti-inflammatory mechanism of IMP by inducing an AD response in keratinocytes through TNF-α/IFN-γ or IL-4 stimulation.

**Results:**

Contrary to the prevailing view that IMP is an unhealthy microbial metabolite, we found that IMP-treated AD-like skin lesions mice showed significant improvement in their clinical symptoms, including ear thickness, epidermal and dermal thickness, and IgE levels. Furthermore, IMP antagonized the expansion of myeloid (neutrophils, macrophages, eosinophils, and mast cells) and Th cells (Th1, Th2, and Th17) in mouse skin and prevented mitochondrial reactive oxygen species production by inhibiting mitochondrial energy production. Interestingly, we found that IMP inhibited AD by reducing glucose uptake in cells to suppress proinflammatory cytokines and chemokines in an AD-like *in vitro* model, sequentially downregulating the PI3K and mTORC2 signaling pathways centered on Akt, and upregulating DDIT4 and AMPK.

**Discussion:**

Our results suggest that IMP exerts anti-inflammatory effects through the metabolic reprogramming of skin inflammation, making it a promising therapeutic candidate for AD and related skin diseases.

## Introduction

Atopic dermatitis (AD) is a chronic inflammatory skin disease characterized by severe itching, skin dryness, recurrent eczematous lesions, and high susceptibility to infection (1). The disease is accompanied by the activation of the skin’s innate immunity and exaggerated IgE sensitization to environmental allergens, and begins with a Th2 immune response that expands to adaptive immune responses involving Th1 and Th17 (2). The Th2 immune response is the primary cause of skin inflammation in AD (2). Blocking IL-4 and IL-13 receptors produced by Th2 cells has been clinically proven to be safe and effective, and is currently being applied as an alternative treatment for AD (3). Furthermore, phase II and III clinical trials of various biological and small-molecule antagonists targeting signal transduction pathways involved in the Th2 immune response are underway to develop therapies for AD (3).

Activation, proliferation, and differentiation of T helper cells are highly dependent on energy production and synthesis of metabolites (4). CD4^+^ T cells are characterized by a preference for energy production via the mitochondrial pathway (5). Th2 cell differentiation requires extensive metabolic reprogramming in response to changes in intracellular metabolic requirements and nutrient exposure, which are primarily regulated by the mammalian target of rapamycin (mTOR) signaling (6). The mTOR complex integrates cytokine receptor signaling while regulating glucose, amino acid, and lipid metabolism (6). This requires the upregulation of glucose uptake (6). Previous studies have highlighted the importance of mTOR complex 2 (mTORC2; an mTOR complex) in Th2 cell differentiation in allergic diseases (7). mTORC2 promotes Th2 cell differentiation through various mechanisms, and as the Th2 cells enter inflamed tissue sites, they continue to differentiate through exposure to various inflammatory cytokines in the tissues (6). It also regulates reactive oxygen species (ROS) production and respiration in mitochondria (8). However, it is not yet clear how mTORC2 deregulation affects AD.

The recent discovery of disease-associated small microbiome molecules has led to a growing interest in their potential role in pathogenesis and as therapeutics (9, 10). Imidazole propionate (IMP) is a histidine metabolite produced by the gut microbiota (11). Elevated IMP levels have been associated with intestinal inflammation, cardiovascular disease, and an impaired glucose response in individuals with type 2 diabetes (11). IMP is also associated with the inhibition of insulin signaling through mTORC1 in the mTOR complex in mice and humans (12). Accumulating evidence suggests that imidazole and its derivatives possess a variety of pharmacological properties, including antifungal, antibacterial, anti-inflammatory, antiviral, and anticancer properties (13). However, studies on IMP are limited, and its effects on skin diseases have not been studied.

In this study, we investigated whether IMP suppresses skin inflammation in AD by regulating mTORC2 signaling and mitochondrial ROS. We found that IMP attenuated AD-associated inflammation and severity in both *in vivo* and *in vitro* models. These results indicate that IMP represents a potentially useful candidate for the treatment of Th2-driven inflammatory diseases, in addition to AD.

## Results

### IMP ameliorates the severity of AD-like skin lesions in a mouse model

**Figure 1:**
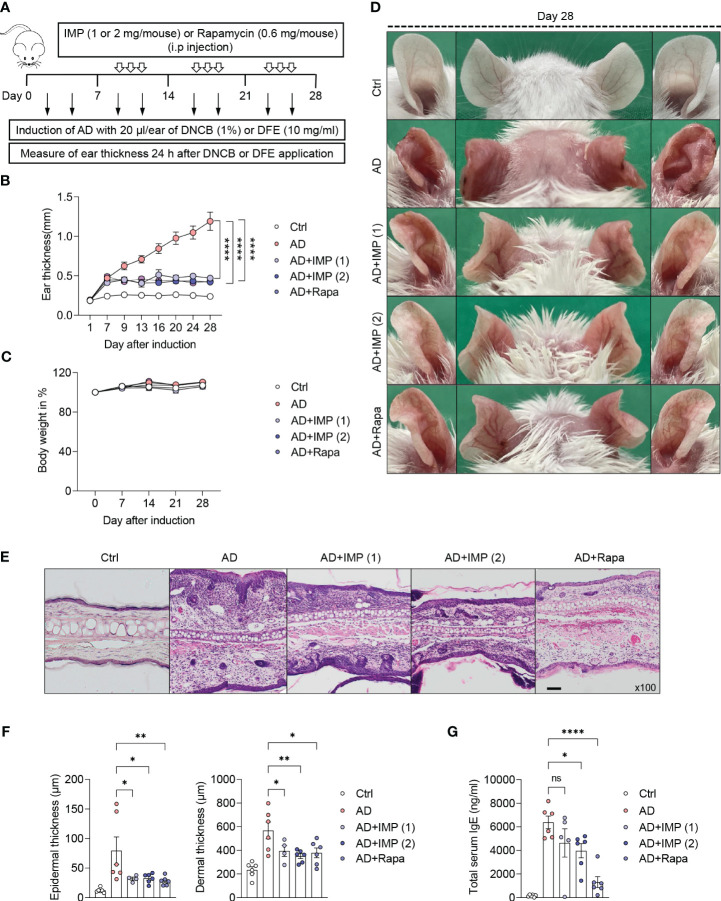
IMP attenuates the clinical symptoms of AD-like skin lesions in a mouse model. BALB/c mice were induced with DNCB and DFE, and treated with IMP (1 or 2 mg/mouse) or Rapa (0.6 mg/mouse). **(A)** Experimental design for the induction of AD-like skin lesions. The mice (n = 4–6/group) were divided into five groups. **(B)** Ear thickness was measured 24 h after DNCB or DFE application using a dial thickness gauge. **(C)** Mouse body weight was calculated as a percentage of the initial weight. **(D)** Images of the mouse ears from each representative group on day 28. **(E)** Representative photomicrographs of ear sections stained with hematoxylin and eosin (H&E) (×100 magnification; scale bar = 20 µm). **(F)** Epidermal and dermal thicknesses were measured using microphotographs of H&E-stained ear tissues. **(G)** Serum IgE levels were analyzed using ELISA. Data are presented as the mean ± standard error of mean (SEM). ****p < 0.0001, **p < 0.01, and *p < 0.05 indicate significant reduction compared to the AD group. Ctrl, control; AD, atopic dermatitis; DNCB, 2,4-dinitrochlorobenzene; DFE, *Dermatophagoides farina* extract; IMP, imidazole propionate; Rapa, rapamycin. ns: no significant.

In the present study, we investigated the potential therapeutic effects of IMP on AD-like skin inflammation using an AD-like skin lesion model. We used *Dermatophagoides farinae* extract (DFE) and 1-chloro-2,4 dinitrochlorobenzene (DNCB) to induce AD in the ear lobes of BALB/c mice by treating them for 28 days. Ear thickness was measured 24 h after the application of DFE or DNCB for AD-like skin lesions induction. IMP and Rapa were injected intraperitoneally every other day, three times a week, from day 7 to day 28 after induction of AD-like skin lesions. mTOR inhibitor Rapa was used as a positive control to investigate the possibility that IMP could inhibit the mTOR pathway in AD pathogenesis. The schematic diagram of AD-like skin lesions induction and IMP treatment are shown in **Figure 1A**. Disease severity was assessed by measuring the thickness, imaging, and histological analysis of the ear lobes. The ear thickness started to increase in the AD-like skin lesion mice 7 days after AD-like skin lesions induction, but was significantly reduced in the IMP-treated group (**Figure 1B**). The size and weight of the draining lymph nodes (auricular LNs) were also significantly reduced in the IMP-treated AD-like skin lesions group (**Supplementary Figure S2**). No change in body weight was observed in any of the groups following IMP administration (**Figure 1C**). Images of the mouse ears showed an inflammatory response with erythema, edema, keratinization, and scaling. These symptoms were ameliorated in the IMP-treated group (**Figure 1D**). Histopathological evaluation revealed significant thickening of the epidermis and dermis in AD-like skin lesion mice, which was significantly decreased in the AD-like skin lesion mice treated with IMP and Rapa (**Figures 1E, F**). In AD-like skin lesions, elevated IgE levels are associated with disease severity and IgE levels can be used to monitor treatment response (3). Our results showed that serum IgE levels were significantly lower in AD-like skin lesion mice treated with IMP (2 mg/mouse) than those in untreated AD-like skin lesion mice (**Figure 1G**). These findings suggest that IMP effectively mitigates the severity of AD-like skin lesions.

### IMP suppresses the expansion of inflammatory myeloid cells in the skin of AD-like skin lesion mice

**Figure 2:**
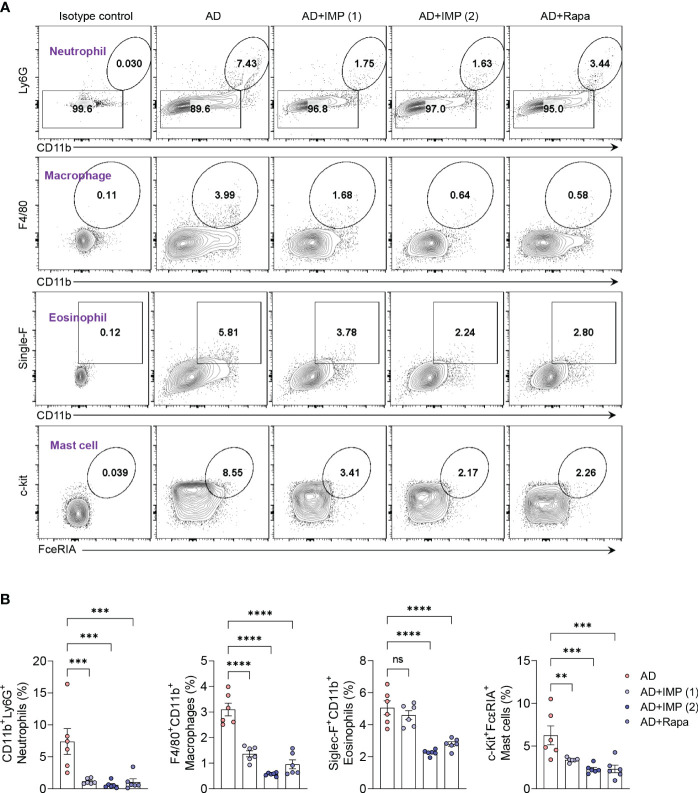
IMP inhibits the inflammatory myeloid cells in the skin of AD mice. **(A, B)** Representative flow cytometry plots and bar charts show percentage of CD11b^+^Ly6G^+^ (neutrophils), F4/80^+^CD11b^+^ (macrophages), single-F^+^CD11b^+^ (eosinophils), and c-Kit^+^FcεRIA^+^ (mast cells) in the ear skin cells (n = 6/group). Data are presented as the mean ± SEM. ****p < 0.0001, ***p < 0.001, and **p < 0.01 indicate significant reduction compared to the AD group. AD, atopic dermatitis; IMP, imidazole propionate; Rapa, rapamycin. ns: no significant.

Although Th2 cell-driven inflammatory responses are important in AD, various immune cells other than Th2 cells participate in the pathophysiology of AD. Myeloid cells (neutrophils, macrophages, eosinophils, and mast cells) play crucial roles in pruritus, barrier damage, and inflammation in AD (14). These pro-inflammatory myeloid cells are rare in normal skin but increase during inflammation, and their inhibition is associated with disease resolution (15, 16). Therefore, we isolated skin cells from the ear tissues of AD-like skin lesion mice and determined the inhibitory effects of IMP and Rapa on myeloid cells. Flow cytometric analysis showed that the frequency of myeloid cell infiltration in the skin was significantly reduced in a dose-dependent manner by the recruitment of neutrophils with the CD11b^+^Ly6G^+^ phenotype and macrophages with the CD11b^+^F4/80^+^ phenotype in the IMP-treated AD-like skin lesion mice compared to that in the AD-like skin lesion mice. Moreover, significant inhibition of mast cell expansion, characterized by eosinophils with the single-F^+^CD11b^+^ phenotype and mast cells with c-kit^+^FcεRIA^+^ phenotype, was also observed in AD-like skin lesion mice treated with IMP and Rapa compared to that in the untreated AD-like skin lesion mice (**Figure 2**). These results suggest that IMP markedly inhibits inflammatory cell infiltration in skin tissue.

### IMP inhibits the expansion of Th1/Th2/Th17 cell immune responses and induces the expression of Foxp3^+^ regulatory T cells in the skin of AD-like skin lesion mice

**Figure 3:**
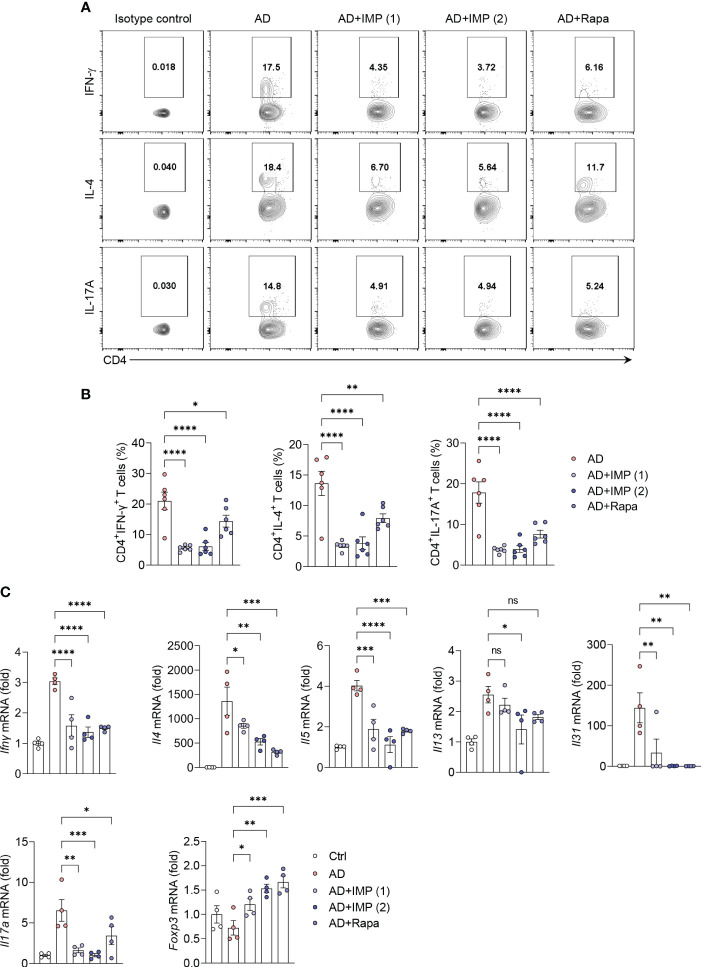
IMP regulates the T cell immune response in the inflamed skin of AD mice. **(A, B)** Representative flow cytometry plots and bar graphs showing percent CD4^+^ T cells expressing IFN-γ, IL-4 or IL-17A in the ear skin cells (n = 6/group). **(C)** Gene expression of Th1 (*Ifnγ*), Th2 (*Il4*, *Il5*, *Il13*, and *Il31*), and Th17 (*Il17a*) cytokines and Treg (*Foxp3*) transcription factor in the ears of AD and IMP or Rapa-treated AD mice. To determine the cytokine expression in mice, the ears were excised on day 28. Gene expression was analyzed using real-time PCR (n = 4/group). The gene expression levels were normalized to that of β-actin. Data are presented as the mean ± SEM. ****p < 0.0001, ***p < 0.001, **p < 0.01, and *p < 0.05 indicate significant reduction compared to the AD group. Ctrl, control; AD, atopic dermatitis; IMP, imidazole propionate; Rapa, rapamycin. ns: no significant.

The acute onset of AD is characterized by infiltration of Th2 cells. In contrast, Th1 cells can be detected in chronic AD lesions as early as within an hour post-onset, alongside Th2 and Th17 cells (17). Therefore, CD4+ T helper cells are central to inducing AD, as highlighted in numerous studies, clinical trials, and mechanistic analyses, making them pivotal in the development of new therapeutic strategies (17). We isolated cells from the skin of AD and IMP-treated AD-like skin lesion mice, and investigated their antagonistic effects on Th1, Th2, and Th17 immune responses using intracellular cytokine staining. We found that the number of Th1 (CD4^+^IFN-γ^+^), Th2 (CD4^+^IL-4^+^), and Th17 (CD4^+^IL-17A^+^) cells were significantly reduced in the skin cells of both IMP- and Rapa-treated AD-like skin lesion mice, whereas this was not observed in AD-like skin lesion mice treated with Rapa alone (**Figures 3A, B**). We then analyzed the RNA expression of key cytokines produced by Th1, Th2, Th17, and Treg cells in the skin tissues. As predicted, the mRNA levels of cytokines produced by Th1 (*Ifnγ*), Th2 (*Il4*, *Il5*, *Il13*, and *Il31*), and Th17 (*Il17a*) were markedly reduced in the skin of IMP-treated AD-like skin lesion mice, providing clear evidence that IMP antagonizes T-cell responses during AD onset (**Figure 3C**). As Foxp3 has been reported to suppress T cell-mediated inflammatory responses in an AD-like skin lesions mouse model (18), we checked the RNA expression of Foxp3 to determine whether the IMP-mediated attenuation of Th1, Th2, and Th17 cells in AD was partially because of regulatory cells. We found that the expression of *Foxp3* was increased in the IMP-treated AD-like skin lesions group (**Figure 3C**).

### IMP alters energy metabolism and inhibits the production of mitochondrial ROS in the skin of AD-like skin lesion mice

**Figure 4:**
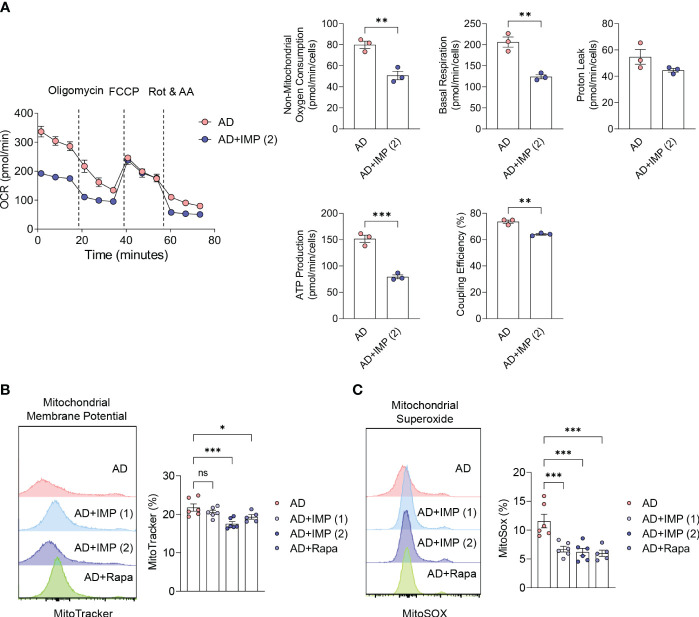
IMP inhibits mitochondrial ROS production by altering the inflammatory metabolic profile in AD skin. **(A)** Real-time changes in the oxygen consumption rate (OCR) of skin cells in response to oligomycin, FCCP, and Rot/AA. The bar charts show the mitochondrial oxygen consumption, basal and maximal respiratory capacity, proton leak, ATP production rate and coupling efficiency. Representative histograms and bar graphs showing total mitochondrial mass (n = 3/group). **(B)** and mitochondrial ROS production **(C)**. Mitochondrial mass and ROS were analyzed by flow cytometry in skin cells labeled with MitoTracker green or MitoSOX red (n = 6/group). Data are presented as the mean ± SEM. ***p < 0.001, **p < 0.01, and *p < 0.05 indicate significant reduction compared to the AD group. AD, atopic dermatitis; IMP, imidazole propionate; Rapa, rapamycin; OCR, oxygen consumption rate; FCCP, carbonyl cyanide-p-trifluoromethoxyphenylhydrazone; Rot/AA, rotenone and antimycin A; ATP, adenosine triphosphate; ROS, reactive oxygen species. ns: no significant.

Several physiological factors are involved in the severity and progression of AD, and measurement of energy metabolism and oxidative stress in the inflamed skin represent important markers for assessing the inflammatory state (19). In human skin inflammation, energy metabolism is highly dependent on the oxidative phosphorylation (OXPHOS) pathway (19). In this study, we extracted mouse ear skin cells 28 days after AD induction and combined the cells to measure the OCR, a key OXPHOS parameter. We found that basal and maximal OCR, mitochondrial respiratory capacity, ATP production rate, and ATP synthesis efficiency via oxygen consumption (coupling efficiency) were reduced in IMP-treated AD-like skin lesion mice compared to those in untreated AD-like skin lesion mice (**Figure 4A**). To understand how the mitochondrial metabolic profile changes under the influence of IMP, we investigated mitochondrial function in AD-like skin lesions mouse skin cells using MitoTracker™. Accumulation of dysfunctional mitochondria was observed in AD-like skin lesions mouse skin without IMP treatment, but this was reduced in the IMP- and Rapa-treated groups (**Figure 4B**). MitoSOX™, a mitochondria-specific ROS indicator, was used to measure mitochondrial ROS levels, and the assay revealed that ROS levels were increased in AD-like skin lesion mice, but decreased in the IMP- and Rapa-treated AD-like skin lesion groups (**Figure 4C**). These results suggest that the activated state of skin inflammatory cells in AD is associated with changes in metabolic processes, and that IMP inhibits mitochondrial dysfunction and ROS production.

### IMP inhibits glucose uptake in keratinocytes and regulates mTORC2 signaling to suppress inflammatory cytokines and chemokines

Next, we investigated the effects and mechanisms of IMP in an *in vitro* AD model. Keratinocytes in AD patients can amplify the recruitment and production of Th2 cell cytokines by releasing chemokines such as CCL17 and CCL22 (20). TNF-α has been reported to promote the production of CCL17 and CCL22 in keratinocytes, inducing features similar to atopic dermatitis (21, 22). In HaCaT cells, the levels of CCL17 and CCL22 are enhanced upon stimulation with TNF-α and IFN-γ (22, 23). Indeed, skin lesions in AD also show increased expression of CCL17 or CCL22 in response to elevated TNF-α and IFN-γ, and this has been used in numerous studies as an *in vitro* model mimicking AD (22, 24, 25). Based on these findings, we decided to apply this mechanism as an *in vitro* model for AD. Furthermore, we verified the efficacy of IMP in an AD environment *in vitro* using mouse primary keratinocytes induced with IL-4.

**Figure 5:**
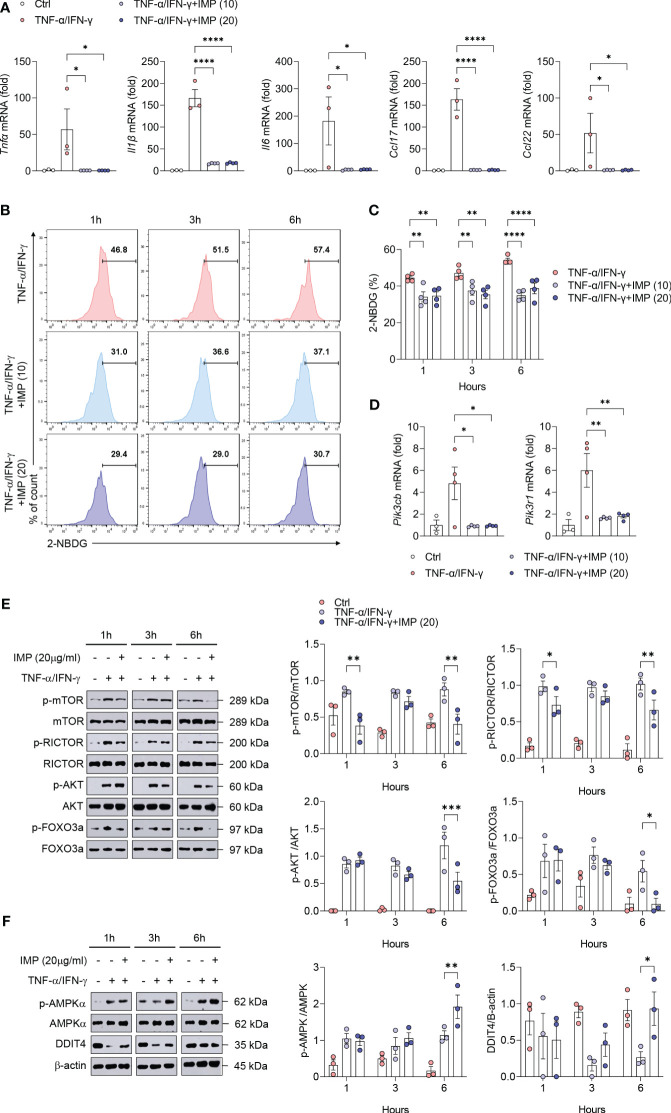
Induction of AMPK/DDIT4 by IMP inhibits mTORC2 signaling and reduces inflammatory cytokines and chemokines in AD *in vitro*. Human keratinocytes HaCaT cells were stimulated with TNF-α (10 ng/mL) and IFN-γ (10 ng/mL) in the presence or absence of IMP (10 or 20 µg/mL) for 6 h or indicated times (n = 3-4/group). **(A)** Gene expression of proinflammatory cytokines (*Tnfα*, *Il1β*, and *Il6*), and chemokines (*Ccl17 and Ccl22*) was analyzed using real-time PCR. **(B, C)** Representative histograms and bar graphs showing glucose uptake in HaCaT cells, which was determined by incubation with 2-NBDG for 2 h, followed by flow cytometry. **(D)** Gene expression of PI3K signaling molecules (*Pik3cb* and *Pik3r1*) was analyzed using real-time PCR. Protein expression of mTORC2 targets **(E)**, and that of AMPK and DDIT4 **(F)** in HaCaT cells stimulated in the presence or absence of IMP (20 μg/mL) was determined by western blotting. Data are presented as the mean ± SEM. ****p < 0.0001, ***p < 0.001, **p < 0.01, and *p < 0.05 indicate significant reduction compared to TNF-α/IFN-γ. IMP, imidazole propionate; 2-NBDG, 2-deoxy-2-[(7-nitro-2,1,3-benzoxadiazol-4-yl)amino]-D-glucose; mTOR, mammalian target of rapamycin; mTORC2, mammalian target of rapamycin complex 2; Rictor, rapamycin-insensitive companion of mammalian target of rapamycin; AKT, protein kinase B; FOXO3a, forkhead box O3a; AMPK, AMP-activated protein kinase; DDIT4, DNA-damage-inducible transcript 4.

HaCaT cells were treated with different concentrations of IMP (5, 10, and 20 µg/mL) for 24 h to determine the effective concentration, and cell viability was measured using the MTT assay. The results showed that none of the concentrations of IMP tested affected the survival of HaCaT cells following 24 h of treatment (**Supplementary Figure S3**). Moreover, in the *in vitro* AD models using HaCaT cells or mouse primary keratinocytes stimulated with TNF-α/IFN-γ or IL-4, IMP treatment suppressed the gene expression of proinflammatory cytokines (TNF-α, IL-1β, and IL-6) and chemokines (CCL17 and CCL22) (**Figure 5A** and **Supplementary Figure S4A**). Based on the results of our *in vivo* experiments, we hypothesized that the inhibition of skin inflammation is related to glucose degradation. To test this hypothesis, HaCaT cells were stimulated with TNF-α/IFN-γ for 1, 3, and 6 h, and mouse primary keratinocytes were stimulated with IL-4 for 12, 24, and 48 h, respectively. Following treatment with IMPs, the intracellular glucose uptake status was determined by flow cytometry using the fluorescently-labeled glucose uptake molecule, 2-NBDG. The results showed an increase in glucose uptake at all time points for both TNF-α/IFN-γ-stimulated HaCaT cells and IL-4-stimulated mouse primary keratinocytes. In the IMP-treated group, glucose uptake was significantly reduced at all time points (**Figure 5B, C** and **Supplementary Figures S4B, C**). As an important metabolic regulator, mTOR significantly affects glucose metabolism (26). Therefore, we sought to determine whether IMP alters the global glucose metabolic pathway and modulates mTOR signaling. We found that IMP significantly inhibited the PI3Ks genes, *Pik3b* and *Pik3r1*, which encode transcription factors that mediate signals upstream of mTOR (**Figure 5D**). As inhibition of PI3K may affect mTOR signaling, we validated these results by analyzing the expression of downstream signaling targets of mTORC2, the mTOR complex related to the Th2 immune response, at various time points after treatment with IMP following stimulation with TNF-α/IFN-γ or IL-4 in HaCaT and mouse primary keratinocytes by western blotting. The results showed that TNF-α/IFN-γ stimulation increased the phosphorylation of mTOR, RICTOR, AKT, and FOXO3a, the downstream targets of mTORC2, whereas IMP impaired their activation (**Figure 5E** and **Supplementary Figure S4D**). AMPK and DDIT4 are negative regulators of mTORC2 and suppress Th2-mediated allergic immune responses (7, 27). Therefore, to investigate how IMP inhibits mTORC2 signaling, the activation status of AMPK and DDIT4 was determined by western blotting. We found that the phosphorylation of AMPK remained unchanged after 1 h of IMP treatment, but increased in the IMP-treated group, compared to TNF-α/IFN-γ alone, at 3 and 6 h. Similarly, activation of DDIT4 increased following 3 and 6 h of IMP-treatment (**Figure 5F**).

## Discussion

Microbial metabolites play key roles in various tissues and organs in the human body (28). These metabolites act on receptors at various sites, such as the intestine, liver, and central nervous system, and may contribute to metabolic diseases, such as obesity, type 2 diabetes, and cardiovascular disease (28). Microbially produced IMP has been found to be present at higher levels in the plasma of patients with prediabetes and type 2 diabetes. It acts as a negative regulator of type 2 diabetes through the p38/p62/mTORC1 pathway, and inhibits the glucose-lowering effects of metformin (12, 29). However, the role of IMP has not been investigated in skin diseases. In the present study, we found that IMP exerts anti-inflammatory effects on the skin, which is contrary to the findings of previous studies. Based on these findings, we sought to further investigate the role of IMP in skin inflammation, especially in AD, to evaluate the potential medical application of this microbial metabolite.

AD is characterized by intense pruritus, eczema, erythema, swelling, peeling, exudation, and scab formation on the skin, with epidermal barrier abnormalities, T cell-induced skin inflammation, and an increased IgE response in most patients (30). In contrast to the severe skin inflammation observed in AD mice, we found that AD mice treated with IMP showed attenuated disease severity, including inhibition of inflammatory cell infiltration in skin tissue and a significant reduction in serum IgE levels, suggesting that IMP attenuated the disease. AD is a chronic inflammatory skin disease caused by the complex interplay of immune responses (30). In particular, the interaction between myeloid and T cells has a major impact on the onset and progression of AD. In our study, we found that the expansion of neutrophils, macrophages, eosinophils, and mast cells was inhibited in the skin of AD-like skin lesion mice after IMP administration. This suggests that IMP reduces the ability of myeloid cells in the skin to present antigens to Th1/Th2/Th17 T cells, and inhibits T cell activation and subsequent cytokine production, which may impede the persistence and progression of AD inflammation. It is also worth noting that the suppression of Th1/Th2/Th17-produced cytokines and attenuation of inflammation in the skin tissues of IMP-treated AD-like skin lesion mice may also be correlated with the increased expression of Treg-expressing Foxp3 following IMP treatment.

In AD, the inflammatory response of Th cells is activated compared with that of naïve T cells, and promotes the inflammatory response by distorting the direction of OXPHOS metabolism in the mitochondria (31). In our study, the treatment of AD-like skin lesion mice with IMP decreased the OXPHOS activity in the skin. These data suggest that IMP reverses inflammation-related intracellular metabolic programs in T cells. Thus, IMP treatment decreased cytokine expression in Th1, Th2, and Th17 cells, whereas it was associated with increased Foxp3 expression in regulatory T cells.

Mitochondria play a crucial role in skin physiology (31). Their metabolic activity regulates keratinocyte differentiation by generating ROS (31, 32). However, excessive ROS production leads to mitochondrial dysfunction due to an increase in silver mitochondrial mass, which is associated with inflammatory signal activation and cell death (33). We found that treatment with IMP or Rapa reduced the accumulation of dysfunctional mitochondria in skin cells of the AD-like skin lesions mouse model and inhibited ROS production in these mitochondria. These findings suggest that mitochondrial accumulation and ROS production in skin cells are associated with AD pathogenesis and that IMP may directly improve mitochondrial function in AD-induced skin inflammatory cells.

Proliferating cells are more dependent on glucose uptake for growth than quiescent cells (34). Impaired glucose uptake contributes to skin damage and inflammation-related inhibition of keratinocyte proliferation, and is considered a novel strategy for the treatment of skin diseases (34). Previous reports have shown that IMP is significantly increased in patients with type 2 diabetes and impairs glucose tolerance and insulin signaling when administered to mice, suggesting that it acts as a negative regulator (12). However, impaired glucose uptake in the inflamed skin environment is associated with suppression of inflammation (35). Our data showed that in an *in vitro* model of AD-like skin inflammation, IMP treatment resulted in reduced expression of Th2 recruitment chemokines and pro-inflammatory cytokines, and reduced glucose uptake levels (analyzed using 2-NBDG). These results demonstrate that IMP may inhibit inflammatory responses through impaired glucose binding in the AD skin inflammatory milieu, which is in contrast to the results of the limited studies on diseases associated with glucose tolerance and insulin resistance.

Our *in vivo* data showed that Rapa, used as a positive control, modulated mTOR phosphorylation to ameliorate clinical symptoms of AD by reducing epidermal and dermal thickness, inflammatory cell infiltration, and serum IgE and Th1/2 cytokine levels in the skin of AD-like skin lesion mice, which is consistent with previous studies (36). Furthermore, IMP showed similar effects as Rapa, suggesting that it may be directly involved in mTOR signaling in AD. Dysregulation of the PI3Kk/Akt/mTOR pathway is observed in skin cancer, psoriasis, and AD, and is associated with uncontrolled and excessive proliferation of inflammatory skin cells (37). mTOR forms two distinct protein complexes, mTORC1 and mTORC2, which play important roles in T-cell differentiation, skin morphology, and epidermal development (38). mTORC1 promotes Th17 differentiation, Foxp3 expression, and suppresses Treg cell status (39). mTORC2 (Rictor) is essential for Th1 and Th2 cell differentiation, and Akt activation (39). In T cells from pediatric AD patients, the PI3K/Akt pathway is abnormally activated (37). Rictor-deficient mice show filaggrin control of epidermal lipid synthesis and keratinization (40). mTORC2 regulates substrates such as forkhead box (Foxo) proteins O1 and O3 by targeting Akt, resulting in cell cycle suppression and regulation of Th1 transcription factors (38, 39). We found that the PI3K/Akt/mTOR pathway was activated in TNF-α/IFN-γ-stimulated HaCaT cells, an *in vitro* model of AD, and IMP-dependently inhibited mTOR, Rictor and Akt, and Forkhead box 3a phosphorylation. However, IMP did not affect the expression of the mTORC1 constituent protein Raptor and p70-S6K (**Supplementary Figure S5**). These results suggest that in skin inflammation, such as chronic AD with increased Th1/Th2 ratios, IMP may play an important role in maintaining immune cell homeostasis via the mTORC2 pathway. We further demonstrated that the activities of AMPK and DDIT4, which act as negative upstream regulators of AKT/mTOR, were increased by IMP. These results suggest that by upregulating AMPK and DDIT4, IMP may regulate the bioenergetic balance and overactivation of AD-associated skin inflammatory cells via mTORC2.

Metabolites are important molecules that drive energy production and conversion *in vivo*. Because of their reactivity, structural properties, and varying concentrations, they can change rapidly during metabolic processes in the body (4, 41). Therefore, some metabolites may participate in pro-inflammatory activities, whereas others may exhibit anti-inflammatory effects (41). Similarly, IMP may play a negative role in metabolic diseases associated with impaired glucose tolerance and insulin resistance; however, its anti-inflammatory effects remain unclear.

Our data revealed the regulatory capacity of IMP on mitochondrial function, ROS production, and mTORC2 activity, suggesting that IMP possesses anti-inflammatory effects and has potential as a novel alternative therapy for the treatment of AD, a T cell-mediated chronic skin inflammatory disease. Given these findings, further research is required to elucidate how metabolites, such as IMP, influence the interactions between the host and microbes in T-cell-driven skin diseases, and explore the potential to reverse their effects in specific disease contexts.

## Materials and methods

### Chemicals

The chemical structure of IMP is shown in **Supplementary Figure S1**. IMP and rapamycin (Rapa) were purchased from Sigma-Aldrich and Selleckchem, respectively.

### Cell culture and stimulation

The human keratinocyte cell line HaCaT was purchased from the CLS Cell Line Service (Eppelheim, Germany). The cells were cultured in Dulbecco’s modified Eagle’s medium (DMEM) supplemented with 10% fetal bovine serum (FBS) and antibiotics (100 U/mL penicillin G and 100 μg/mL streptomycin) at 37°C with 5% CO_2_. To generate an *in vitro* AD model using the HaCaT cells, the cells were treated with 10 ng/mL each of recombinant human TNF-α and IFN-γ (both from R&D Systems). Simultaneously, the cells were treated with IMP (10 or 20 µg/mL).

### Mice

Six-week-old female BALB/c mice were purchased from Narabiotec Co. Ltd. (Seoul, Korea) and housed at 23 ± 2°C and 50 ± 5% humidity. The animal experiments were approved by the Animal Care and Use Committee of Jeonbuk National University Medical School (JBNU 2021-0169) and were conducted in accordance with the institutional guidelines.

### Induction of AD-like skin lesions in mouse ear

The mouse AD-like skin lesions model was induced according to the method described in our previous report (25). A schematic diagram of the experiment is shown in **Figure 1A**. The mice (n = 4 to 6) were randomly divided into five groups and the hair on the surfaces of both earlobes was stripped thrice using surgical tape (Nichiban, Tokyo, Japan). Then, 20 μL of 2,4-dinitrochlorobenzene (DNCB; 1% solution, dissolved in 1:3 acetone:olive oil, Sigma-Aldrich) was applied to both earlobes, followed by application of 20 μL of *Dermatophagoides farinae* extract (DFE; 10 mg/mL, dissolved in PBS plus 0.5% Tween 20, Greer Laboratory Inc.) three days later. One week after the DNCB/DFE induction, the mice were injected intraperitoneally with IMP (1 or 2 mg/mouse) or Rapa (0.6 mg/mouse) thrice/week. All groups except the IMP and Rapa groups were injected with PBS. The ear thickness was measured 24 h after DNCB/DFE application using a dial thickness gauge (Mitutoyo Co., Tokyo, Japan). All mice were euthanized with CO_2_ on day 28, and blood and tissue samples were collected and analyzed.

### Histology

Ear tissue was fixed in 4% formalin and embedded in paraffin. Sections (4 μm thick) of the ear tissue were stained with hematoxylin and eosin (H&E). Epidermal and dermal thickness, as well as inflammatory cell infiltration, were observed under a microscope. The thickness of the epidermis and dermis were measured based on the H&E staining pattern at ×100 magnification.

### Enzyme-linked immunosorbent assay

Serum IgE levels were measured on day 28 after the first induction using an IgE ELISA Kit (BD Biosciences, Franklin Lakes, NJ, USA) following the manufacturer’s instructions.

### Florescence-activated cell sorting analysis

To detect intracellular cytokines in the skin cells, the cells were stimulated with phorbol-12-myristate-13-acetate (PMA; 20 ng/mL)/ionomycin (1 μg/mL) and Golgi Plug (BD biosciences) for 4 h. After stimulation, the cells were stained with Live/Dead™ Fixable Dead Cell dye (Thermo Fisher Scientific; diluted in PBS) for 15 min at room temperature and washed twice with cold PBS. For myeloid cell surface staining, antibodies were diluted 1:200 and incubated for 20 min at 4°C. After incubation, the samples were washed twice and staining buffer (BD Bioscience) was added. For T cell-producing cytokines, CD4 surface staining was performed followed by fixation and permeabilization using a Foxp3 Staining Kit (Thermo Fisher) and staining with IFN-γ, IL-4, IL-17A, and Foxp3 antibodies according to the manufacturer’s protocol. The following antibodies were used: anti-mouse F4/80 (BD bioscience), anti-mouse CD11b (BD bioscience), anti-mouse Ly6G (BD bioscience), anti-mouse CD170 (Siglec-F) (Biolegend), anti-mouse CD117 (BD bioscience), anti-mouse FceR1a (BD bioscience), anti-mouse IFN-γ, anti-mouse IL-4, anti-mouse IL-17A, anti-mouse Foxp3 (Thermo), and isotype control (BD bioscience). All samples were collected using an Attune NxT acoustic focusing cytometer (Thermo Fisher Scientific). Live cells were gated based on FSC-A, SSC-A, and fixable viability live/dead dye, and then acquired with an Attune NxT acoustic focusing cytometer (Thermo Fisher). Data analysis was performed using the FlowJo (ver. 10.7.1) software.

### Real-time quantitative PCR

Total RNA was extracted from cultured cells and frozen tissues using the RNAIso Plus Reagent (Takara, Tokyo, Japan) according to the manufacturer’s instructions. cDNA synthesis and RT-qPCR analyses were performed as described previously (42). The RNA samples were dissolved in RNase-free water (Thermo Fisher Scientific) and purified by phenol/chloroform extraction. The RevertAid™ First Strand cDNA Synthesis Kit (Thermo Fisher Scientific) was used for cDNA synthesis. The cDNA was then mixed with SYBR^®^ Green PCR Master Mix (Applied Biosystems) along with the primers for qPCR. Gene expression of cytokines were analyzed using the StepOnePlus™ Real-Time PCR System (Thermo Fisher) according to the manufacturer-recommended PCR parameters. The primer sequences are listed **Supplementary Table S1**. Target gene expression was determined using the standard comparative ΔΔCt method and normalized to that of the housekeeping gene, β-actin.

### Oxygen consumption rate

Tissue cells isolated from the ear skin were pre-cultured in Seahorse XF RPMI medium (containing 10 mM XF glucose, 2 mM XF glutamine, and 1 mM XF pyruvate) at a density of 5 × 10^5^ cells/well in poly D-lysine-coated XFp cell culture miniplates. The OCR was assessed using the XFp Cell Mito Stress Test Kit (Agilent) following the manufacturer’s instructions and measured using a Seahorse XFp Analyzer (Agilent).

### Glucose uptake assay

Tissue cells from mouse ear skin (5 × 10^5^ cells/well) were seeded in a 96-well plate and incubated with 2-[N-(7-nitrobenz-2-oxa-1,3-diazol-4-yl) amino]-2-deoxy-D-glucose (2-NBDG, 0.01 mg/mL, Thermo Fisher) at 37°C for 30 min. Following incubation, the cells were washed twice with PBS and stained for 15 min at room temperature with Live/Dead™ Fixable Dead Cell dye (Thermo Fisher) to specifically detect the viable cells. The samples were analyzed using an Attune NxT acoustic focusing cytometer (Thermo Fisher).

### Western blotting

Cell lysates were prepared and western blotting was performed as described previously (42). Proteins were separated using 6%, 10%, or 12% sodium dodecyl sulfate-polyacrylamide gel electrophoresis. Proteins were detected using the following primary antibodies: anti-phospho-AMPKα (Cell Signaling Technology, clone 40H9), anti-AMPKα (Cell Signaling Technology), anti-phospho-mTOR (Cell Signaling Technology, clone D9C2), anti-mTOR (Cell Signaling Technology, clone 7C10), anti-β-actin (Cell Signaling Technology, clone 13E5), anti-DDIT4 (Proteintech), anti-phospho-Rictor (Cell Signaling Technology, clone D30A3), anti-Rictor (Cell Signaling Technology, clone 53A2), anti-phospho-FoxO3a (Cell Signaling Technology, clone D18H8), anti-FoxO3a (Cell Signaling Technology, clone D19A7), anti-phospho-AKT (Cell Signaling Technology, clone Ser473), anti-AKT (Cell Signaling Technology, clone Ser473), anti-phospho-p70 S6 Kinase (Thr389) (Cell Signaling Technology, 9205S), anti p70 S6 Kinase (Cell Signaling Technology.9202S), anti-phospho-Raptor(Ser792) (Cell Signaling Technology, 2083S), and Raptor (24C12) (Cell Signaling Technology.2280S) antibodies. After the incubating with the corresponding secondary antibody, the ECL substrate (Thermo Fisher) signals were detected using an Amersham Imager 600 (Cytiva Life Sciences, GE HealthCare).

### Statistical analysis

Statistical analyses were performed using GraphPad Prism software (version 10.0). One-way analysis of variance (ANOVA) was used for the Holm-Šídák *post-hoc* test. Data are presented as mean ± standard error of the mean (SEM). Significance is indicated as follows: *p < 0.05; **p < 0.01; ***p < 0.001; ****p < 0.0001.

## Data availability statement

The original contributions presented in the study are included in the article/**Supplementary Materials**. Further inquiries can be directed to the corresponding author.

## Ethics statement

The animal study was approved by Animal Care and Use Committee of Jeonbuk National University Medical School (JBNU 2021-0169). The study was conducted in accordance with the local legislation and institutional requirements.

## Author contributions

HK: Data curation, Formal Analysis, Visualization, Writing – review & editing. JL: Data curation, Formal Analysis, Visualization, Writing – review & editing. DY: Data curation, Writing – review & editing. HP: Data curation, Writing – review & editing. EC: Data curation, Writing – review & editing. KN: Data curation, Methodology, Writing – review & editing. JP: Data curation, Methodology, Writing – review & editing. JC: Conceptualization, Funding acquisition, Investigation, Project administration, Supervision, Writing – original draft, Writing – review & editing.

## References

[1] Langan SM Irvine AD Weidinger S. Atopic dermatitis. Lancet. (2020) 396:345–60. doi: 10.1016/S0140-6736(20)31286-1 32738956

[2] Bieber T. Atopic dermatitis: an expanding therapeutic pipeline for a complex disease. Nat Rev Drug Discovery. (2022) 21:21–40. doi: 10.1038/s41573-021-00266-6 34417579 PMC8377708

[3] Torres T Ferreira EO Goncalo M Mendes-Bastos P Selores M Filipe P. Update on atopic dermatitis. Acta Med Port. (2019) 32:606–13. doi: 10.20344/amp.11963 31493365

[4] O'Neill LA Kishton RJ Rathmell J. A guide to immunometabolism for immunologists. Nat Rev Immunol. (2016) 16:553–65. doi: 10.1038/nri.2016.70 PMC5001910 27396447

[5] MacIver NJ Michalek RD Rathmell JC. Metabolic regulation of T lymphocytes. Annu Rev Immunol. (2013) 31:259–83. doi: 10.1146/annurev-immunol-032712-095956 PMC3606674 23298210

[6] Stark JM Tibbitt CA Coquet JM. The metabolic requirements of Th2 cell differentiation. Front Immunol. (2019) 10:2318. doi: 10.3389/fimmu.2019.02318 31611881 PMC6776632

[7] Pandit M Timilshina M Gu Y Acharya S Chung Y Seo SU. AMPK suppresses Th2 cell responses by repressing mTORC2. Exp Mol Med. (2022) 54:1214–24. doi: 10.1038/s12276-022-00832-x PMC9440126 35999454

[8] Vlahakis A Lopez Muniozguren N Powers T. Mitochondrial respiration links TOR complex 2 signaling to calcium regulation and autophagy. Autophagy. (2017) 13:1256–7. doi: 10.1080/15548627.2017.1299314 PMC5529078 28324658

[9] Descamps HC Herrmann B Wiredu D Thaiss CA. The path toward using microbial metabolites as therapies. EBioMedicine. (2019) 44:747–54. doi: 10.1016/j.ebiom.2019.05.063 PMC6606739 31201140

[10] Ma Y Liu X Wang J. Small molecules in the big picture of gut microbiome-host cross-talk. EBioMedicine. (2022) 81:104085. doi: 10.1016/j.ebiom.2022.104085 35636316 PMC9156878

[11] Molinaro A Bel Lassen P Henricsson M Wu H Adriouch S Belda E. Imidazole propionate is increased in diabetes and associated with dietary patterns and altered microbial ecology. Nat Commun. (2020) 11:5881. doi: 10.1038/s41467-020-20412-9 33208748 PMC7676231

[12] Koh A Molinaro A Stahlman M Khan MT Schmidt C Manneras-Holm L. Microbially Produced Imidazole Propionate Impairs Insulin Signaling through mTORC1. Cell. (2018) 175:947–61.e17. doi: 10.1016/j.cell.2018.09.055 30401435

[13] Tolomeu HV Fraga CAM. Imidazole: synthesis, functionalization and physicochemical properties of a privileged structure in medicinal chemistry. Molecules. (2023) 28:838. doi: 10.3390/molecules28020838 36677894 PMC9865940

[14] Fania L Moretta G Antonelli F Scala E Abeni D Albanesi C. Multiple roles for cytokines in atopic dermatitis: from pathogenic mediators to endotype-specific biomarkers to therapeutic targets. Int J Mol Sci. (2022) 23:2684. doi: 10.3390/ijms23052684 35269828 PMC8910412

[15] Dominguez PM Ardavin C. Differentiation and function of mouse monocyte-derived dendritic cells in steady state and inflammation. Immunol Rev. (2010) 234:90–104. 20193014 10.1111/j.0105-2896.2009.00876.x

[16] Kasraie S Werfel T. Role of macrophages in the pathogenesis of atopic dermatitis. Mediators Inflamm. (2013) 2013:942375. doi: 10.1155/2013/942375 23533313 PMC3603294

[17] Biedermann T Skabytska Y Kaesler S Volz T. Regulation of T cell immunity in atopic dermatitis by microbes: the yin and yang of cutaneous inflammation. Front Immunol. (2015) 6:353. doi: 10.3389/fimmu.2015.00353 26217343 PMC4500098

[18] Fyhrquist N Lehtimaki S Lahl K Savinko T Lappetelainen AM Sparwasser T. Foxp3+ cells control Th2 responses in a murine model of atopic dermatitis. J Invest Dermatol. (2012) 132:1672–80. doi: 10.1038/jid.2012.40 22402436

[19] Cibrian D de la Fuente H Sanchez-Madrid F. Metabolic pathways that control skin homeostasis and inflammation. Trends Mol Med. (2020) 26:975–86. doi: 10.1016/j.molmed.2020.04.004 32371170

[20] Humeau M Boniface K Bodet C. Cytokine-mediated crosstalk between keratinocytes and T cells in atopic dermatitis. Front Immunol. (2022) 13:801579. doi: 10.3389/fimmu.2022.801579 35464457 PMC9022745

[21] Danso MO van Drongelen V Mulder A van Esch J Scott H van Smeden J. TNF-alpha and Th2 cytokines induce atopic dermatitis-like features on epidermal differentiation proteins and stratum corneum lipids in human skin equivalents. J Invest Dermatol. (2014) 134:1941–50. doi: 10.1038/jid.2014.83 24518171

[22] Vestergaard C Bang K Gesser B Yoneyama H Matsushima K Larsen CG. A Th2 chemokine, TARC, produced by keratinocytes may recruit CLA+CCR4+ lymphocytes into lesional atopic dermatitis skin. J Invest Dermatol. (2000) 115:640–6. doi: 10.1046/j.1523-1747.2000.00115.x 10998136

[23] Xiao T Kagami S Saeki H Sugaya M Kakinuma T Fujita H. Both IL-4 and IL-13 inhibit the TNF-alpha and IFN-gamma enhanced MDC production in a human keratinocyte cell line, HaCaT cells. J Dermatol Sci. (2003) 31:111–7. doi: 10.1016/S0923-1811(02)00149-4 12670721

[24] Horikawa T Nakayama T Hikita I Yamada H Fujisawa R Bito T. IFN-gamma-inducible expression of thymus and activation-regulated chemokine/CCL17 and macrophage-derived chemokine/CCL22 in epidermal keratinocytes and their roles in atopic dermatitis. Int Immunol. (2002) 14:767–73. doi: 10.1093/intimm/dxf044 12096036

[25] Choi JK Jang YH Lee S Lee SR Choi YA Jin M. Chrysin attenuates atopic dermatitis by suppressing inflammation of keratinocytes. Food Chem Toxicol. (2017) 110:142–50. doi: 10.1016/j.fct.2017.10.025 29050978

[26] Laplante M Sabatini DM. mTOR signaling in growth control and disease. Cell. (2012) 149:274–93. doi: 10.1016/j.cell.2012.03.017 PMC3331679 22500797

[27] Coronel L Hackes D Schwab K Riege K Hoffmann S Fischer M. p53-mediated AKT and mTOR inhibition requires RFX7 and DDIT4 and depends on nutrient abundance. Oncogene. (2022) 41:1063–9. doi: 10.1038/s41388-021-02147-z PMC8837532 34907345

[28] Koh A Backhed F. From association to causality: the role of the gut microbiota and its functional products on host metabolism. Mol Cell. (2020) 78:584–96. doi: 10.1016/j.molcel.2020.03.005 32234490

[29] Koh A Manneras-Holm L Yunn NO Nilsson PM Ryu SH Molinaro A. Microbial Imidazole Propionate Affects Responses to Metformin through p38gamma-Dependent Inhibitory AMPK Phosphorylation. Cell Metab. (2020) 32:643–53.e4. doi: 10.1016/j.cmet.2020.07.012 32783890 PMC7546034

[30] Weidinger S Beck LA Bieber T Kabashima K Irvine AD. Atopic dermatitis. Nat Rev Dis Primers. (2018) 4:1. doi: 10.1038/s41572-018-0001-z 29930242

[31] Feichtinger RG Sperl W Bauer JW Kofler B. Mitochondrial dysfunction: a neglected component of skin diseases. Exp Dermatol. (2014) 23:607–14. doi: 10.1111/exd.12484 24980550

[32] Hamanaka RB Chandel NS. Mitochondrial metabolism as a regulator of keratinocyte differentiation. Cell Logist. (2013) 3:e25456. doi: 10.4161/cl.25456 24475371 PMC3891634

[33] Rizwan H Pal S Sabnam S Pal A. High glucose augments ROS generation regulates mitochondrial dysfunction and apoptosis via stress signalling cascades in keratinocytes. Life Sci. (2020) 241:117148. doi: 10.1016/j.lfs.2019.117148 31830478

[34] Zhang Z Zi Z Lee EE Zhao J Contreras DC South AP. Differential glucose requirement in skin homeostasis and injury identifies a therapeutic target for psoriasis. Nat Med. (2018) 24:617–27. doi: 10.1038/s41591-018-0003-0 PMC6095711 29662201

[35] Watson MJ Vignali PDA Mullett SJ Overacre-Delgoffe AE Peralta RM Grebinoski S. Metabolic support of tumour-infiltrating regulatory T cells by lactic acid. Nature. (2021) 591:645–51. doi: 10.1038/s41586-020-03045-2 PMC7990682 33589820

[36] Yang F Tanaka M Wataya-Kaneda M Yang L Nakamura A Matsumoto S. Topical application of rapamycin ointment ameliorates Dermatophagoides farina body extract-induced atopic dermatitis in NC/Nga mice. Exp Dermatol. (2014) 23:568–72. doi: 10.1111/exd.12463 24903639

[37] Mercurio L Albanesi C Madonna S. Recent updates on the involvement of PI3K/AKT/mTOR molecular cascade in the pathogenesis of hyperproliferative skin disorders. Front Med (Lausanne). (2021) 8:665647. doi: 10.3389/fmed.2021.665647 33996865 PMC8119789

[38] Ding X Bloch W Iden S Ruegg MA Hall MN Leptin M. mTORC1 and mTORC2 regulate skin morphogenesis and epidermal barrier formation. Nat Commun. (2016) 7:13226. doi: 10.1038/ncomms13226 27807348 PMC5095294

[39] Lee K Gudapati P Dragovic S Spencer C Joyce S Killeen N. Mammalian target of rapamycin protein complex 2 regulates differentiation of Th1 and Th2 cell subsets via distinct signaling pathways. Immunity. (2010) 32:743–53. doi: 10.1016/j.immuni.2010.06.002 PMC2911434 20620941

[40] Ding X Willenborg S Bloch W Wickstrom SA Wagle P Brodesser S. Epidermal mammalian target of rapamycin complex 2 controls lipid synthesis and filaggrin processing in epidermal barrier formation. J Allergy Clin Immunol. (2020) 145:283–300.e8. doi: 10.1016/j.jaci.2019.07.033 31401286

[41] Zhang Y Chen R Zhang D Qi S Liu Y. Metabolite interactions between host and microbiota during health and disease: Which feeds the other? BioMed Pharmacother. (2023) 160:114295. doi: 10.1016/j.biopha.2023.114295 36709600

[42] Lee JY Lee JH Lim HJ Kim E Kim DK Choi JK. Aminooxy acetic acid suppresses Th17-mediated psoriasis-like skin inflammation by inhibiting serine metabolism. Front Pharmacol. (2023) 14:1215861. doi: 10.3389/fphar.2023.1215861 37649889 PMC10464615

