# Supplementary material for: Imidazole propionate ameliorates atopic dermatitis-like skin lesions by inhibiting mitochondrial ROS and mTORC2
Source: Front Immunol. 2024 Mar 12;15:1324026. doi: 10.3389/fimmu.2024.1324026 (PMC10964488; doi:10.3389/fimmu.2024.1324026)
Supplement: Supplementary file 1 [file DataSheet_1.docx]

Supplementary Information for

**Imidazole propionate ameliorates atopic dermatitis by inhibiting mitochondrial ROS and mTORC2**

Ha Eun Kim, Jong Yeong Lee, Dong-Hoon Yoo, Hyo-Hyun Park, Eun-Ju Choi, Kyung-Hwa Nam, Jin Park and Jin Kyeong Choi

Corresponding author: Jin Kyeong Choi

E-mail: [jkchoi@jbnu.ac.kr](mailto:jkchoi@jbnu.ac.kr)

**Files includes:**

Methods

Figures S1 to S3

Table S1

**Methods**

**Cell viability**

Cell viability was measured by 3-(4,5-dimethylthiazol-2-yl)-2,5-diphenyltetrazolium bromide (MTT) assay. 5×10^4^ cells were seeded in 96-well plates and treated with various concentrations of IMP for 24 h. Then, 5mg/ml of MTT was added to each well and incubated for 2 h. Formazan crystals were dissolved with DMSO. Results were expressed as percentage absorbance compared to control.

**Mouse primary Keratinocytes**

BALB/c female tail samples were prepared as follows: the tail was cut into three parts, each part was then soaked in 100 μl of Dispase (4 mg/ml) mixed with 9.9 ml of media, and left overnight in a 4°C refrigerator. After shaking at room temperature, the keratinocytes and their surrounding skin were removed, followed by the addition of 10 ml of 0.25% Trypsin-EDTA to 15 ml of 1x PBS. Subsequently, 2.5 ml of 0.25% Trypsin-EDTA and 10 ml of media mix were added to extract the cells. The media used consisted of Gibco keratinocyte SFM with BFE (bovine pituitary extract), EGF, antibiotics (100 μg/mL penicillin G and 100 μg/mL streptomycin), and 0.06M CaCl2.

**Supplementary Fig. S1.**

**
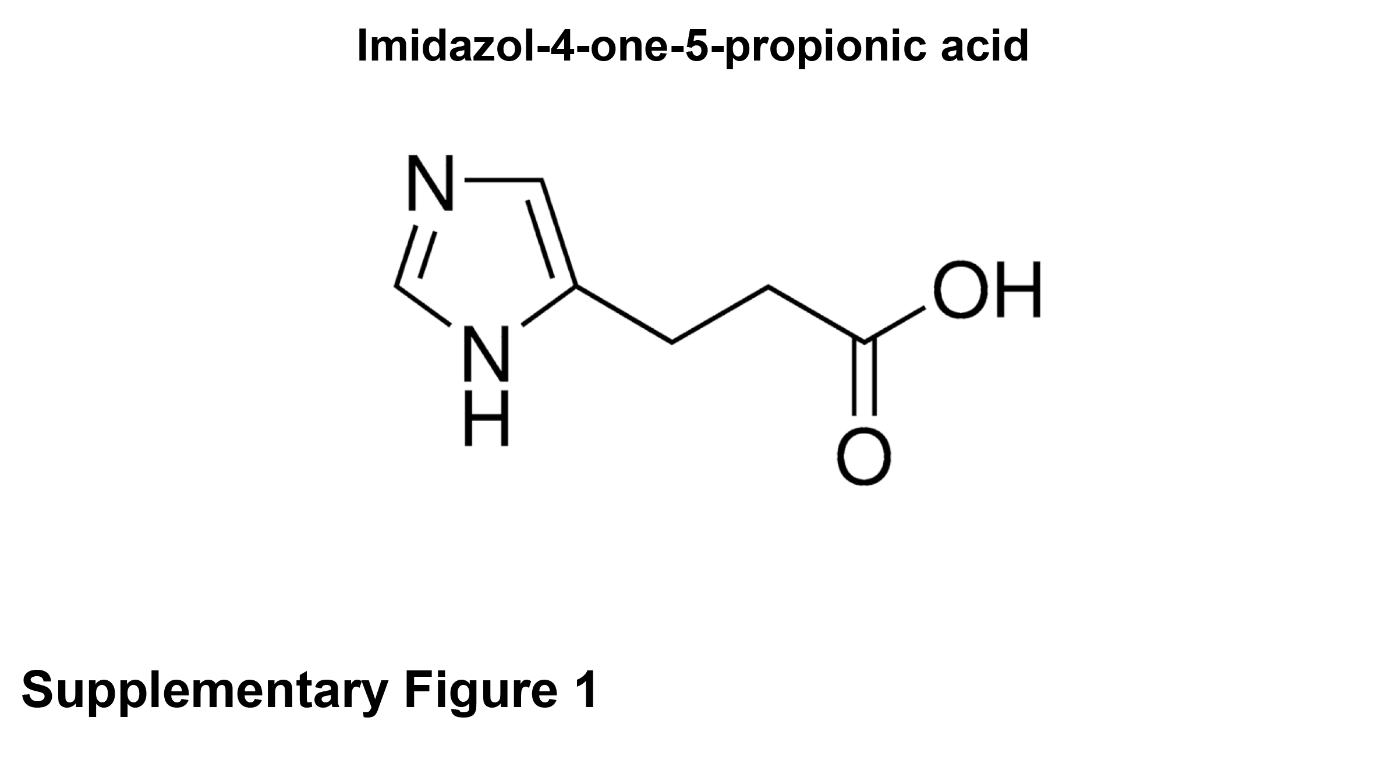
**

**Fig. S1.** Chemical structure of imidazole propionate.

**Supplementary Fig. S2.**

**
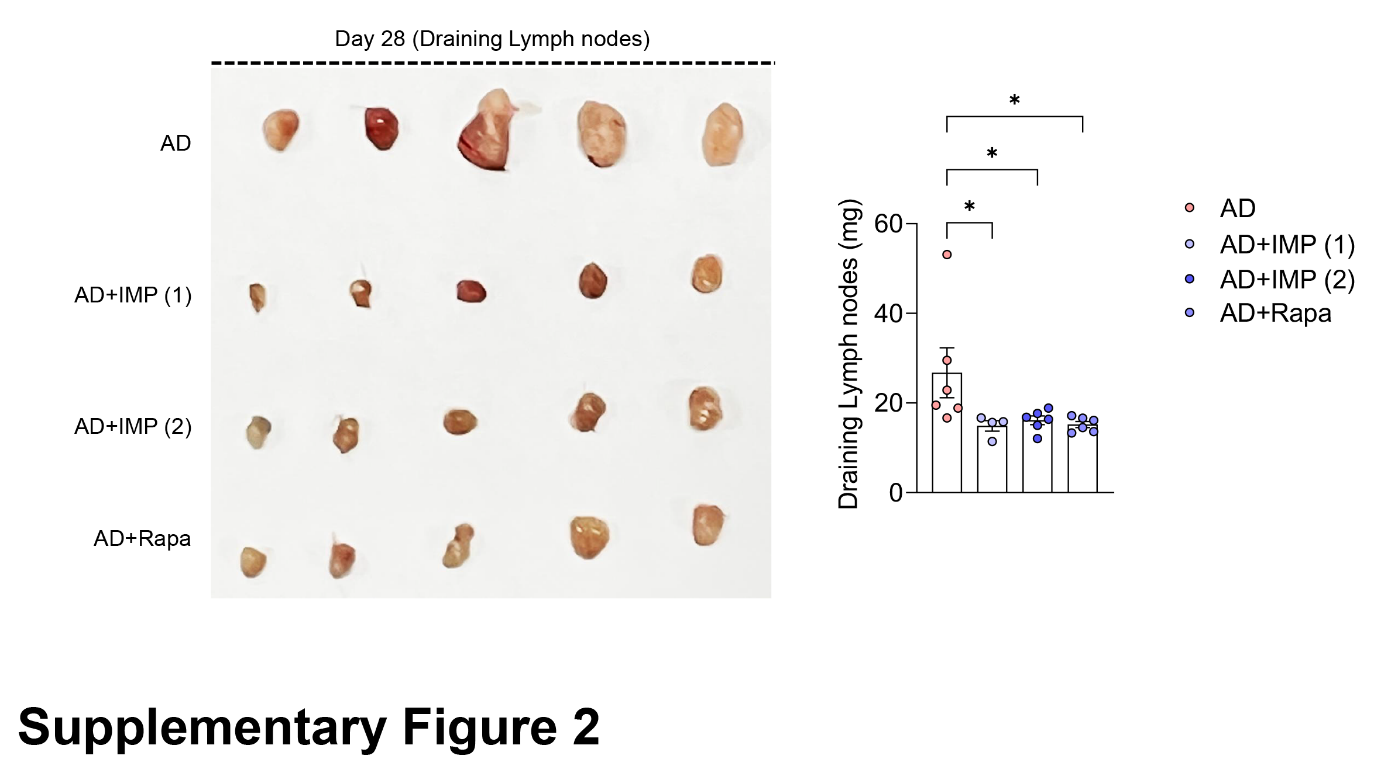
**

**Fig. S2.** Images and weights of the draining lymph nodes from each mouse in the groups obtained on day 28. Data are presented as the mean ± SEM. *p < 0.05 indicates significant reduction compared to the AD group.

**Supplementary Fig. S3.**


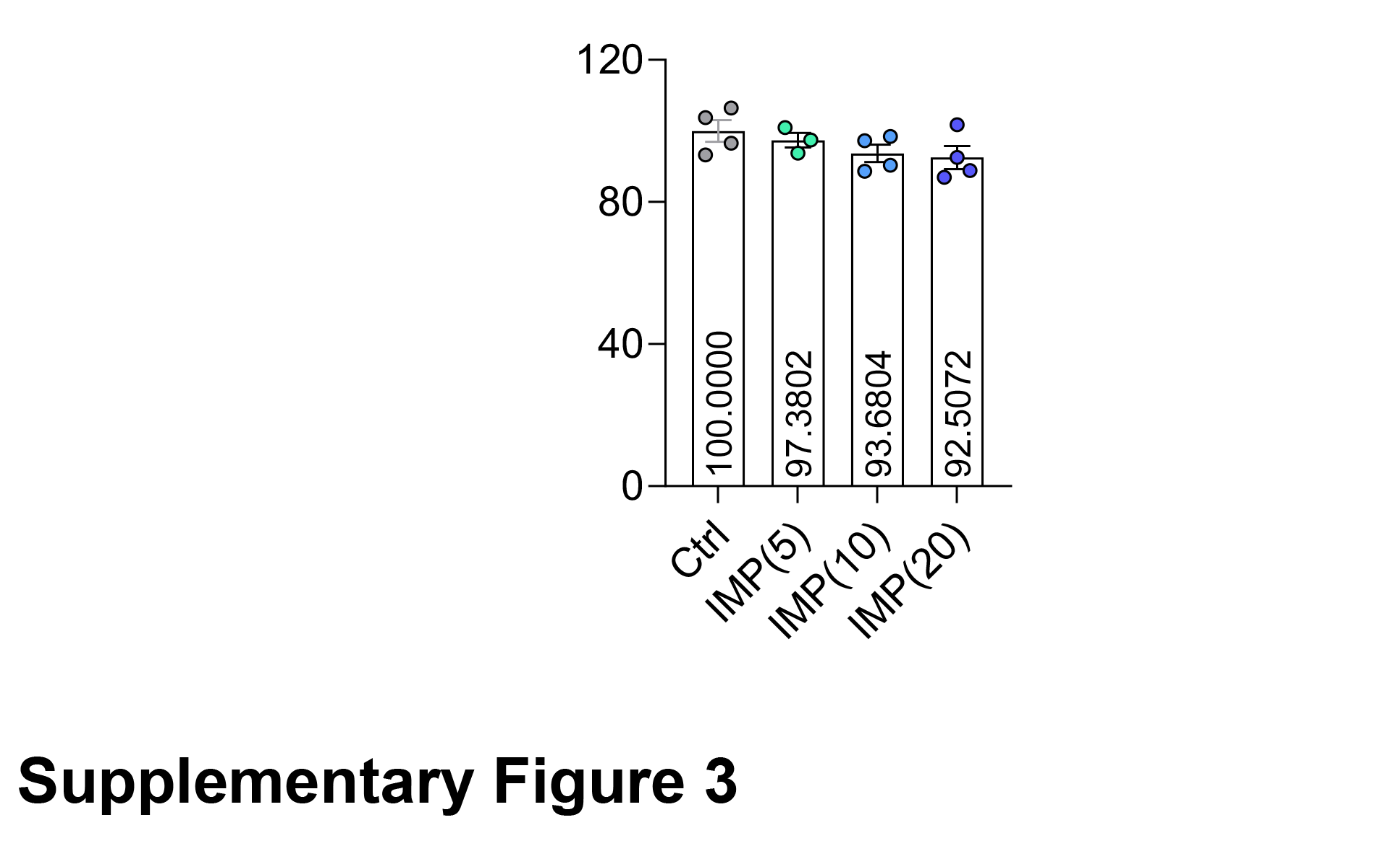


**Fig. S3.** Cell viability of human keratinocytes after IMP treatment. 5 × 10^4^ HaCaT cells were seeded in 96-well plates and treated with various concentrations of IMP for 24 h. Cell viability was assessed using the MTT assay. The data are showed as the mean ± SEM. IMP, imidazole propionate; SEM, standard error of the mean; HaCaT; human keratinocytes.

**Supplementary Fig. S4.**


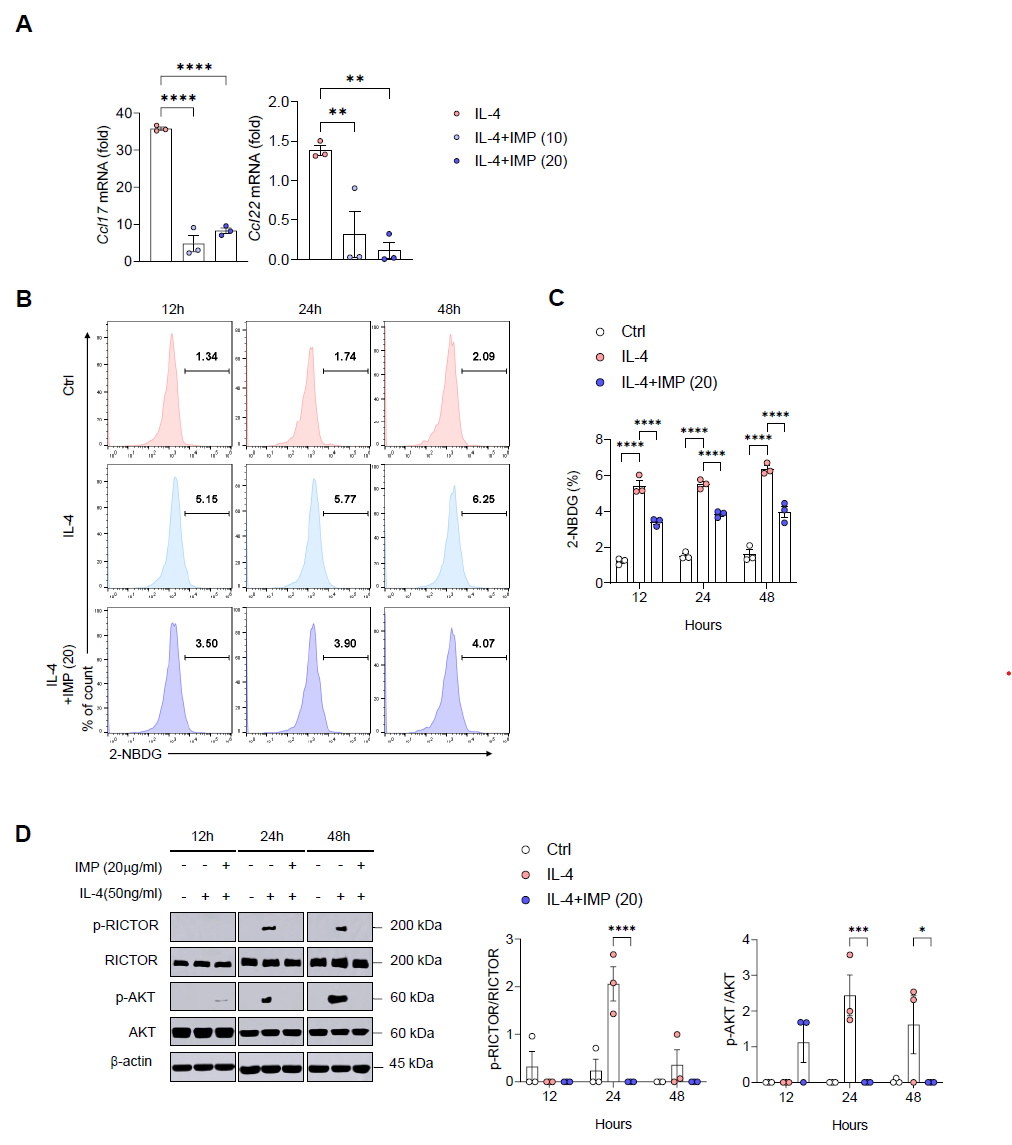


**Fig. S4. Inhibitory effects of IMP on inflammation via mTORC2 regulation in mouse primary keratinocytes.** (A) IMP inhibits gene expression of *Ccl17* and *Ccl22* in mouse primary keratinocytes. Mouse primary keratinocytes were stimulated with IL-4 (50 ng/mL) in the presence or absence of IMP (10 or 20 µg/mL) for 48h (n = 3/group). Gene expression of chemokines (*Ccl17* and *Ccl22*) was analyzed using real-time PCR. (B and C) Representative histograms and bar graphs showing glucose uptake in mouse primary keratinocytes, which was determined by incubation with 2-NBDG for 2 h, followed by flow cytometry. (D) Protein expression of mTORC2 targets in mouse primary keratinocytes stimulated with IL-4 (50 ng/mL) in the presence or absence of IMP (20 μg/mL) was determined by western blotting. Data are presented as the mean ± SEM. ****p < 0.0001, and **p < 0.01 indicate significant reduction compared to IL-4.

**Supplementary Fig. S5.**


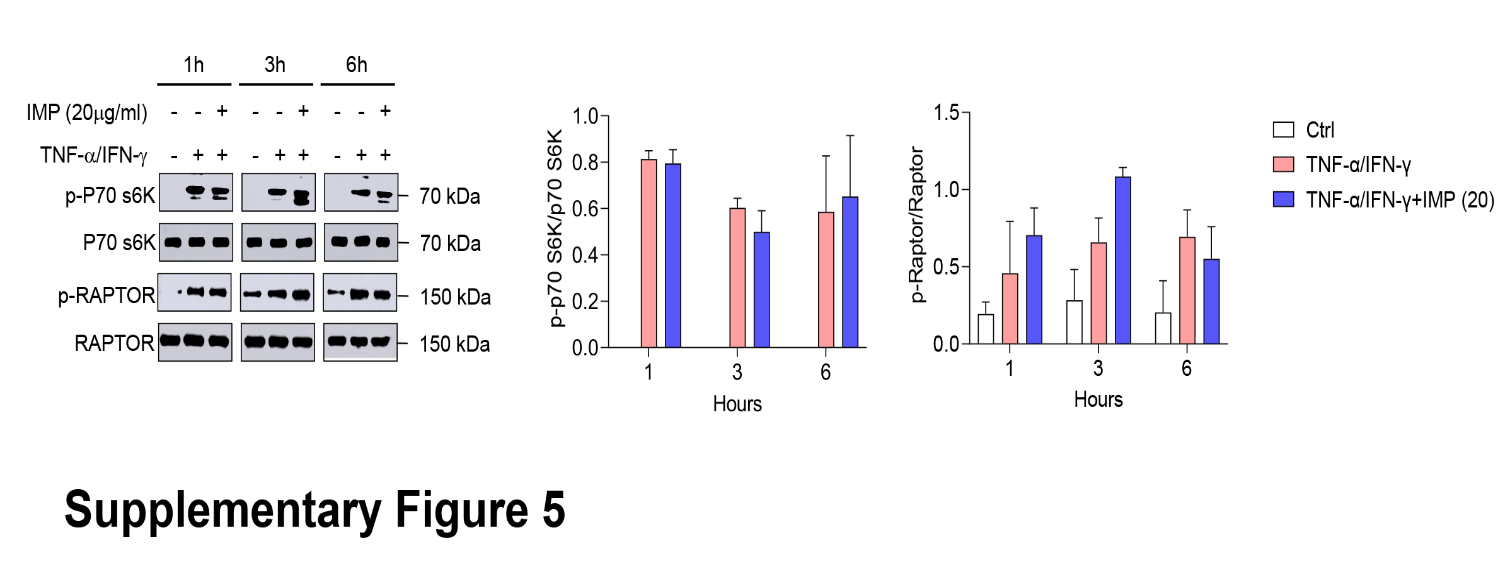


**Fig. S5.** IMP did not affect mTORC1 pathways. Protein expression of mTORC1 targets (p70 S6K and Raptor) in HaCaT cells stimulated with TNF-α (10 ng/mL) and IFN-γ (10 ng/mL) in the presence or absence of IMP (20 μg/mL) was determined by western blotting. Data are presented as the mean ± SEM. Data represent two independent experiments.

**Table S1.** Primer sequences for real-time PCR analysis

Human

| Gene | Forward primer (5’>3’) | Reverse primer (5’>3’) |
| --- | --- | --- |
| *Ccl17* | ACAAGGGGATGGGATCTCCCTCAC | ACTGCTCCAGGGATGCCATCGTTT |
| *Ccl22* | AGGACAGAGCATGGATCGCCTACAGA | TAATGGCAGGGAGGTAGGGCTCCT |
| *Tnfα* | GAGCTGAGAGATAACCAGCTGGTG | CAGATAGATGGGCTCATACCAGGG |
| *IL1β* | GCTGATGGCCCTAAACAGATGAA | TGAAGCCCTTGCTGTAGTGGTG |
| *Pik3cb* | GGTAATCGGAGGATAGGGCAGT | CGGCAGTATGCTTCAAGGATGAC |
| *Pik3r1* | CGCCTCTTCTTATCAAGCTCGTG | GAAGCTGTCGTAATTCTGCCAGG |
| *β-actin* | AGAGCTACGAGCTGCCTGAC | AGCACTGTGTTGGCGTACAG |

Mouse

| Gene | Forward primer (5’>3’) | Reverse primer (5’>3’) |  |
| --- | --- | --- | --- |
| *Ifnγ* | TCAAGTGGCATAGATGTGGAAGAA | TGGCTCTGCAGGATTTTCATG |  |
| *Il17a* | CTCAAAGCTCAGCGTGTCCAAACA | TATCAGGGTCTTCATTGCGGTGGA |  |
| *Foxp3* | GCGAAAGTGGCAGAGAGGTA | GAGGAGCTGCTGAGATGTGA |  |
| *Il4* | ATCATCGGCATTTTGAACGAGGTC | ACCTTGGAAGCCCTACAGACGA |  |
| *Il5* | GAAGTGTGGCGAGGAGAGAC | GCACAGTTTTGTGGGGTTTT | |
| *Il13* | GGGACATGGTTTGCTGCCTA | AGACAGGAGTGTTGCTCTGG | |
| *Il31* | ACACCGAGTTGGAGAGCCGTAT | CTGTCCTCAGACCGATGTTCTC | |
| *Cc17* | CGAGAGTGCTGCCTGGATTACT | GGTCTGCACAGATGAGCTTGCC | |
| *Ccl22* | GTGGAAGACAGTATCTGCTGCC | AGGCTGCGGCAGGATTTTGAG | |
| *βactin* | ACCCTAAGGCCAACCGTGAA | ATGGCGTGAGGGAGAGCATAG | |
