# Supplementary material for: Imidazole propionate ameliorates atopic dermatitis-like skin lesions by inhibiting mitochondrial ROS and mTORC2
Source: Front Immunol. 2024 Mar 12;15:1324026. doi: 10.3389/fimmu.2024.1324026 (PMC10964488; doi:10.3389/fimmu.2024.1324026)

Figure 5E

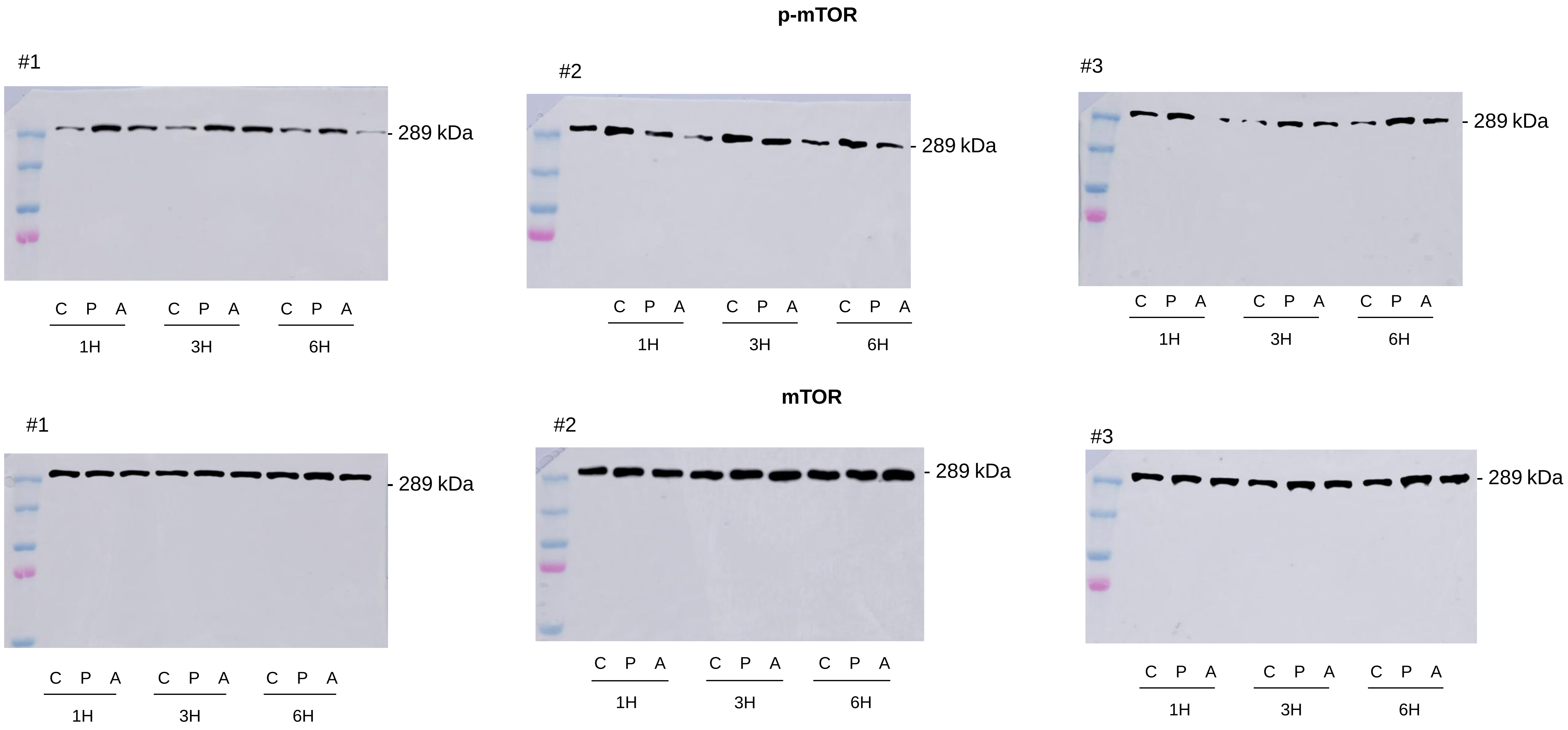

C: Ctrl P: TNF-α(10ng/ml) + IFN-γ(10ng/ml) A: TNF-α(10ng/ml) + IFN-γ(10ng/ml)+ IMP (20㎍/㎖)

Figure 5E

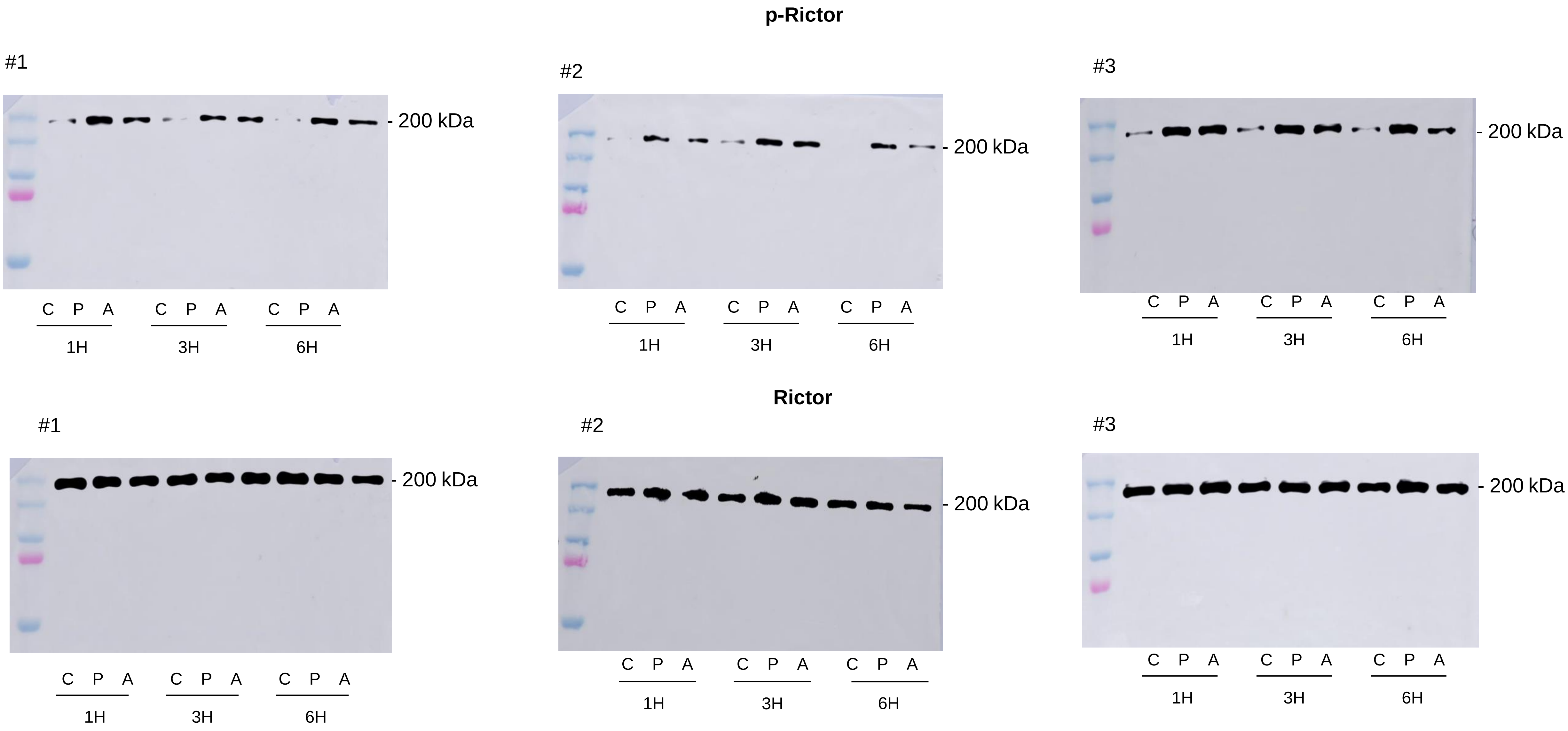

C: Ctrl P: TNF-α(10ng/ml) + IFN-γ(10ng/ml) A: TNF-α(10ng/ml) + IFN-γ(10ng/ml)+ IMP (20μg/mℓ)

Figure 5E

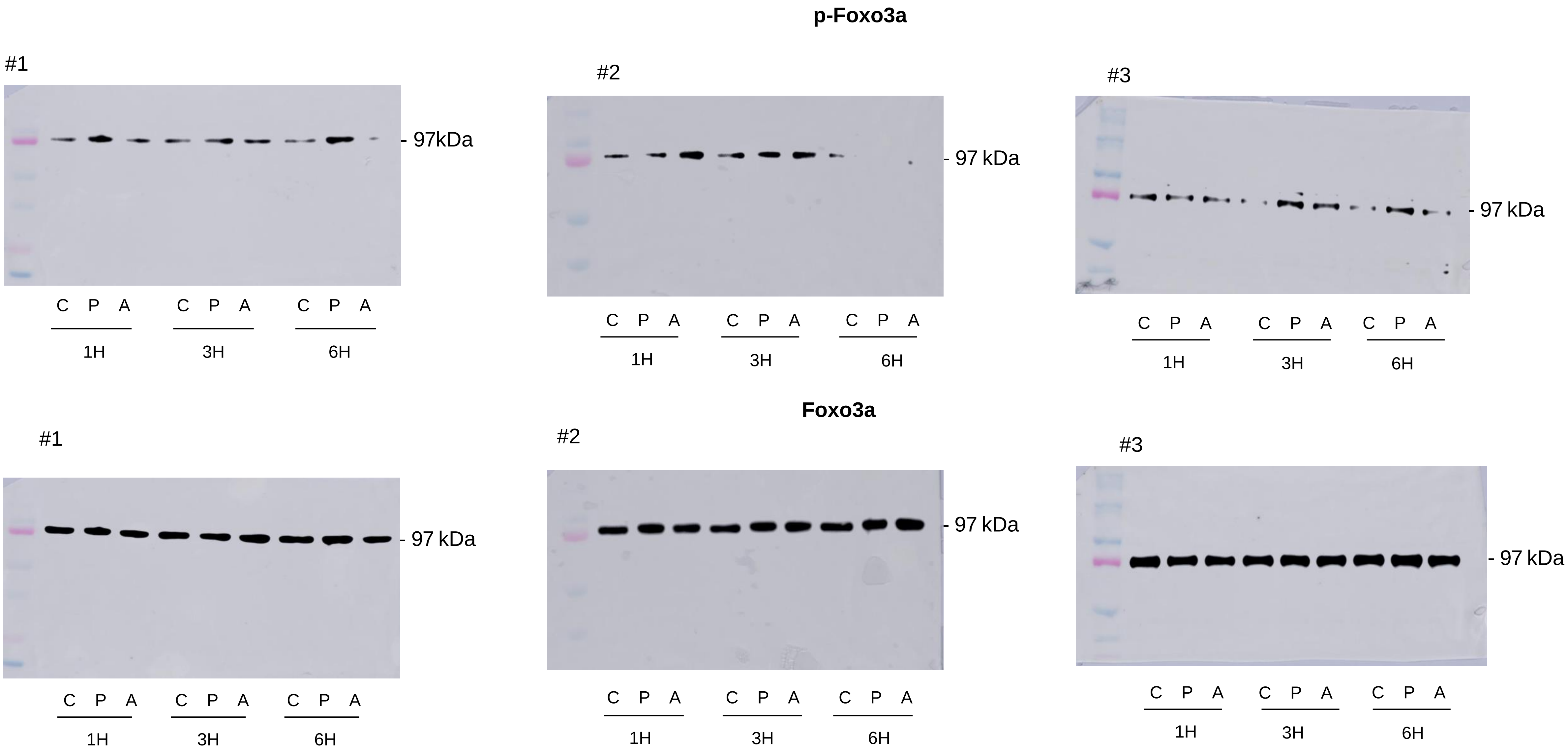

C: Ctrl P: TNF-α(10ng/ml) + IFN-γ(10ng/ml) A: TNF-α(10ng/ml) + IFN-γ(10ng/ml)+ IMP (20㎍/㎖)

Figure 5E

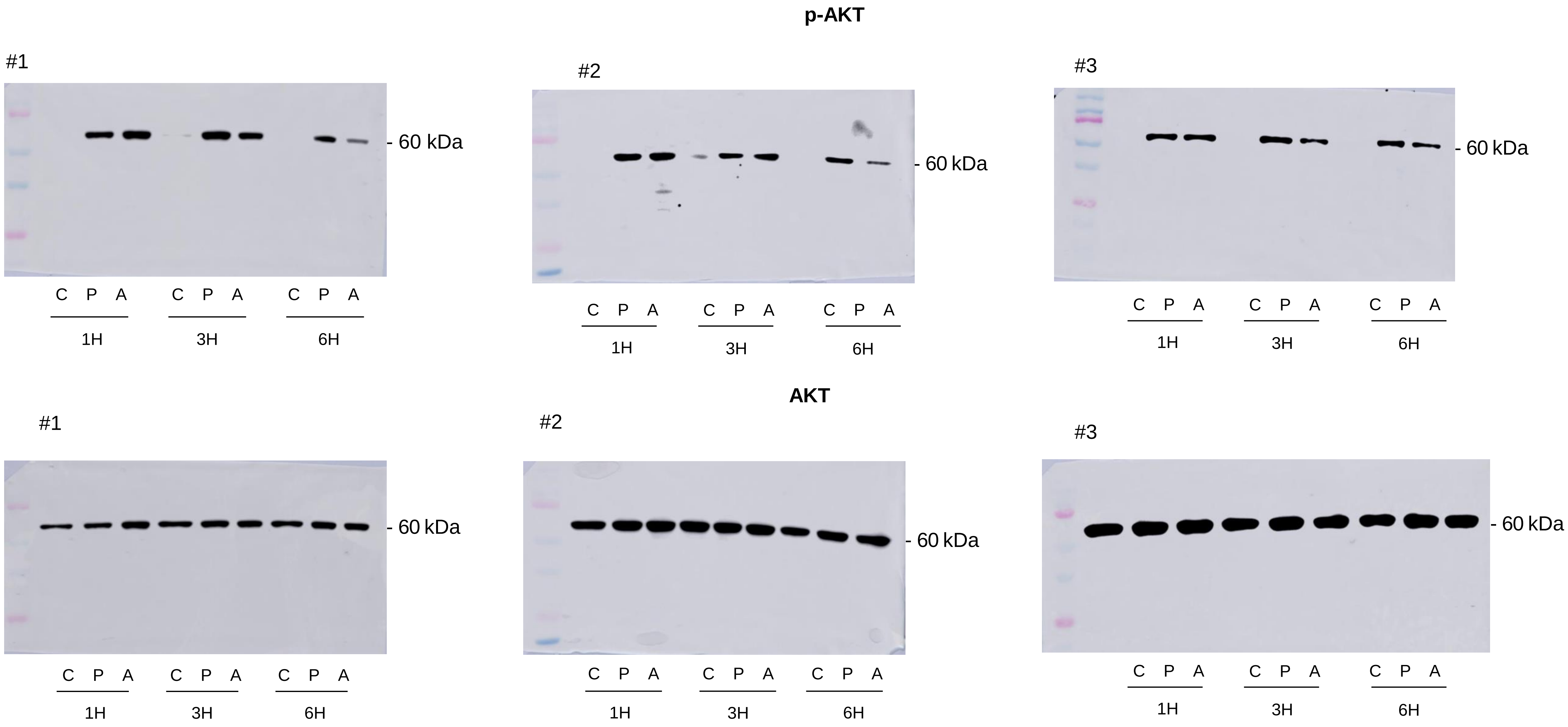

C: Ctrl P: TNF-α(10ng/ml) + IFN-γ(10ng/ml) A: TNF-α(10ng/ml) + IFN-γ(10ng/ml)+ IMP (20μg/mℓ)

Figure 5F

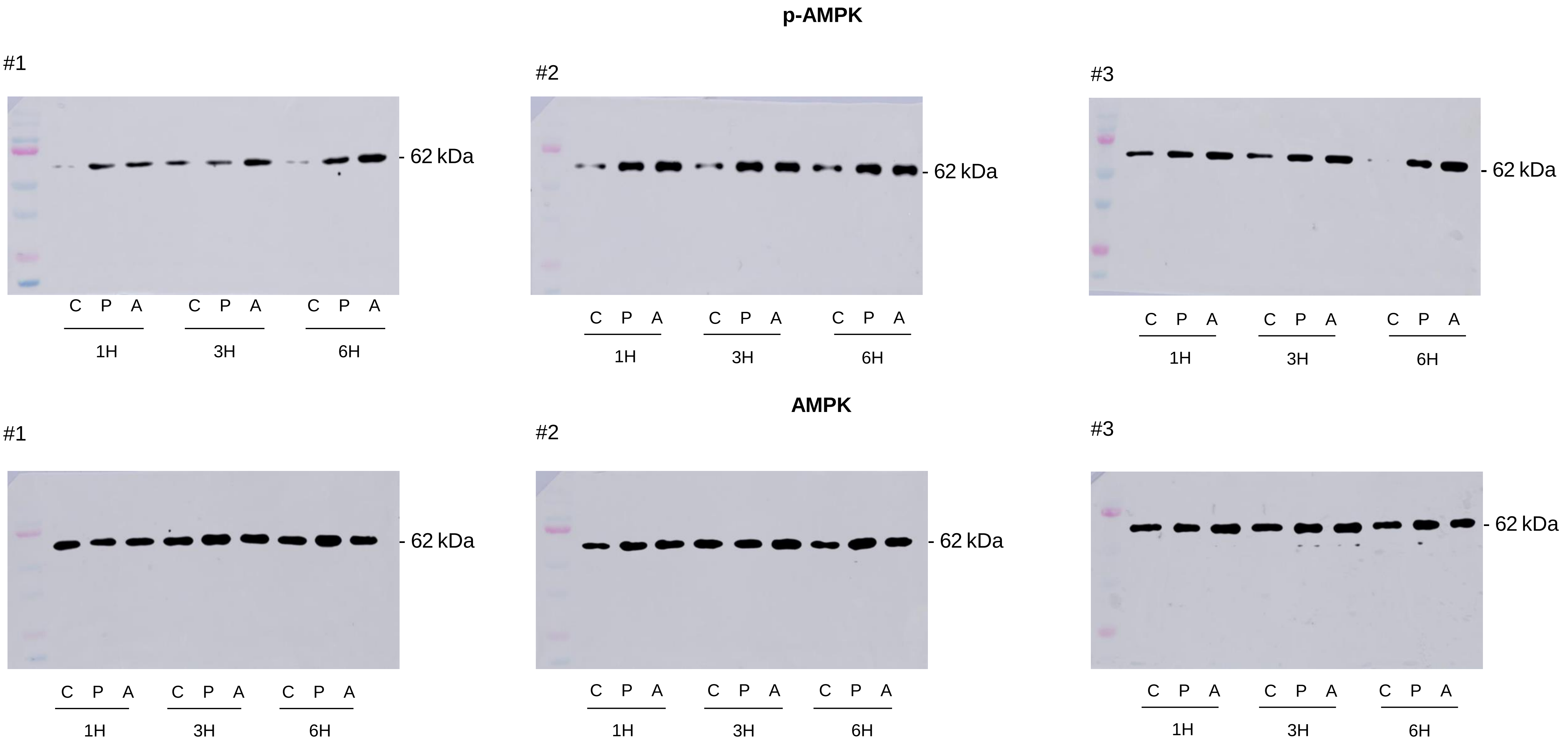

C: Ctrl P: TNF-α(10ng/ml) + IFN-γ(10ng/ml) A: TNF-α(10ng/ml) + IFN-γ(10ng/ml)+ IMP (20㎍/㎖)

Figure 5F

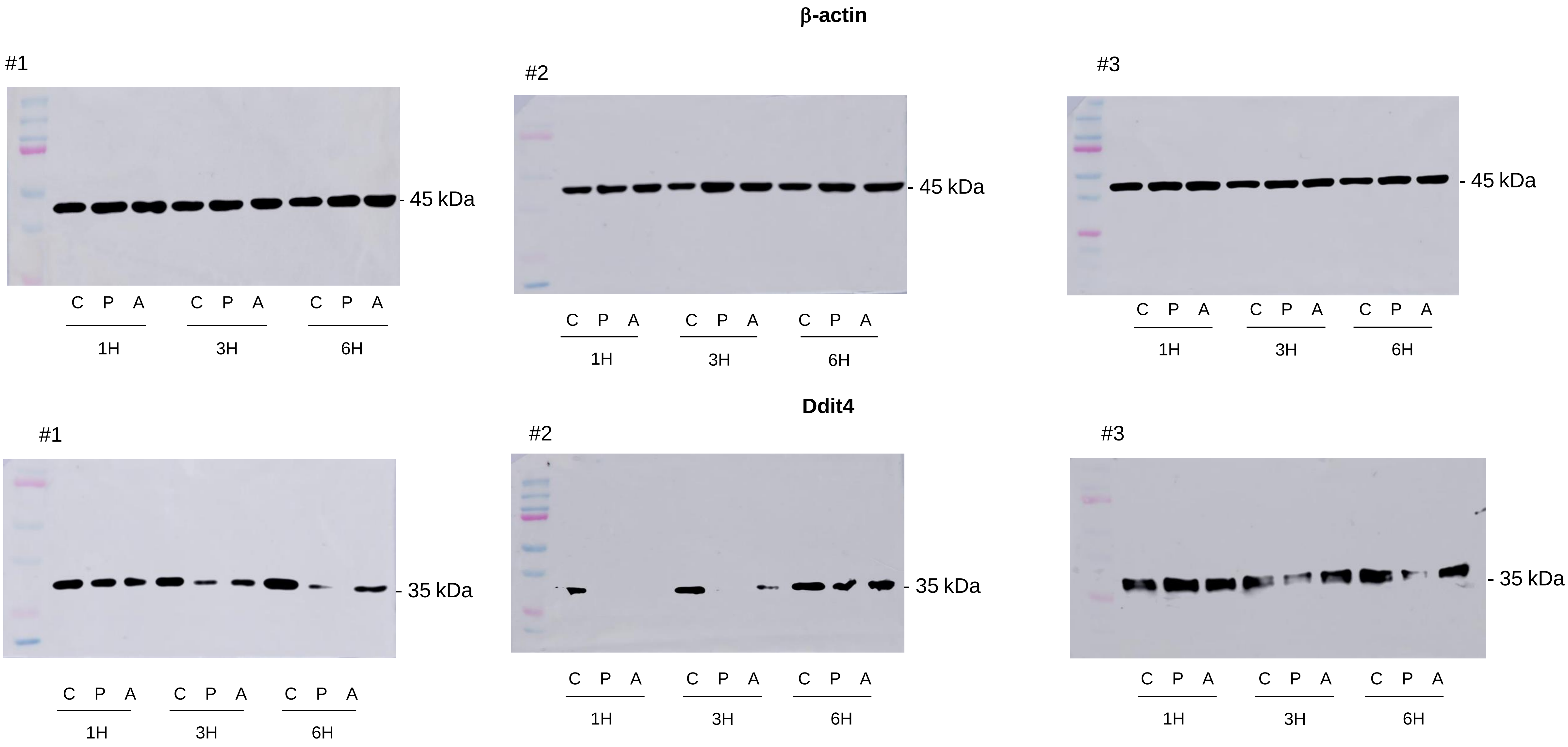

C: Ctrl P: TNF-α(10ng/ml) + IFN-γ(10ng/ml) A: TNF-α(10ng/ml) + IFN-γ(10ng/ml)+ IMP (20㎍/㎖)

Supplementary Figure S5

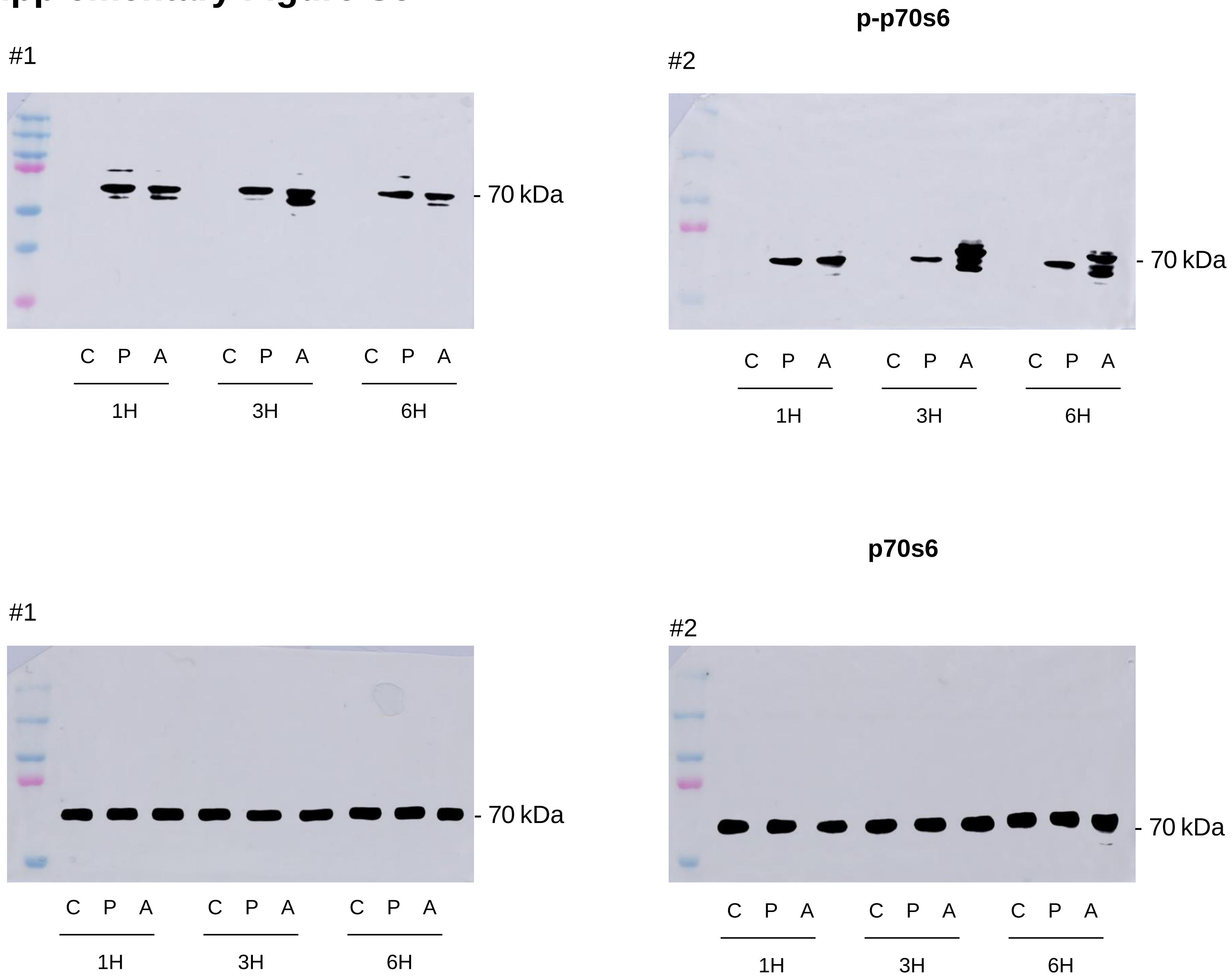

C: Ctrl   P: TNF-α(10ng/ml) + IFN-γ(10ng/ml)   A: TNF-α(10ng/ml) + IFN-γ(10ng/ml)+ IMP (20㎍/㎖)

Supplementary Figure S5

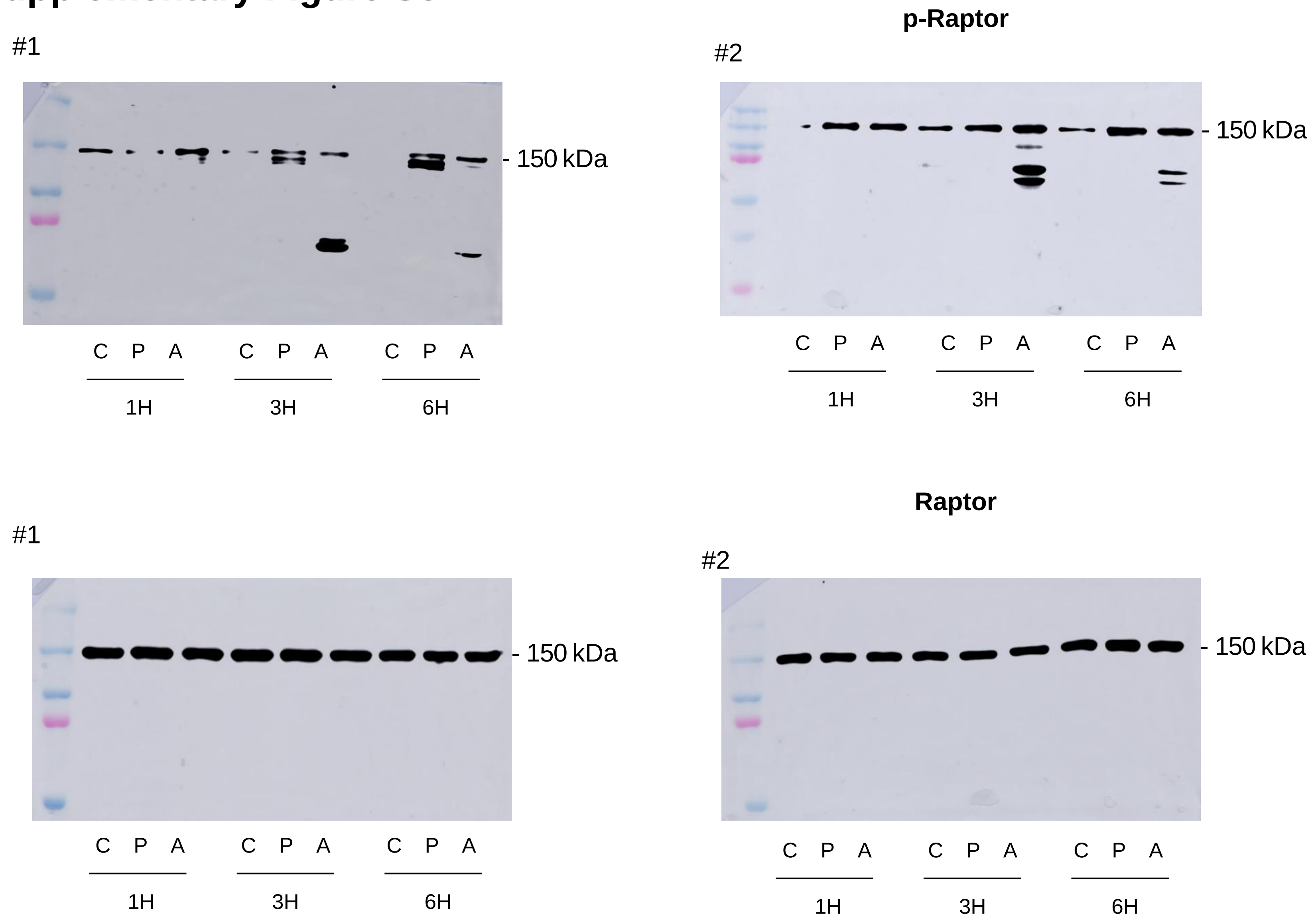

C: Ctrl P: TNF-α(10ng/ml) + IFN-γ(10ng/ml) A: TNF-α(10ng/ml) + IFN-γ(10ng/ml)+ IMP (20㎍/㎖)

Supplementary Figure S4D

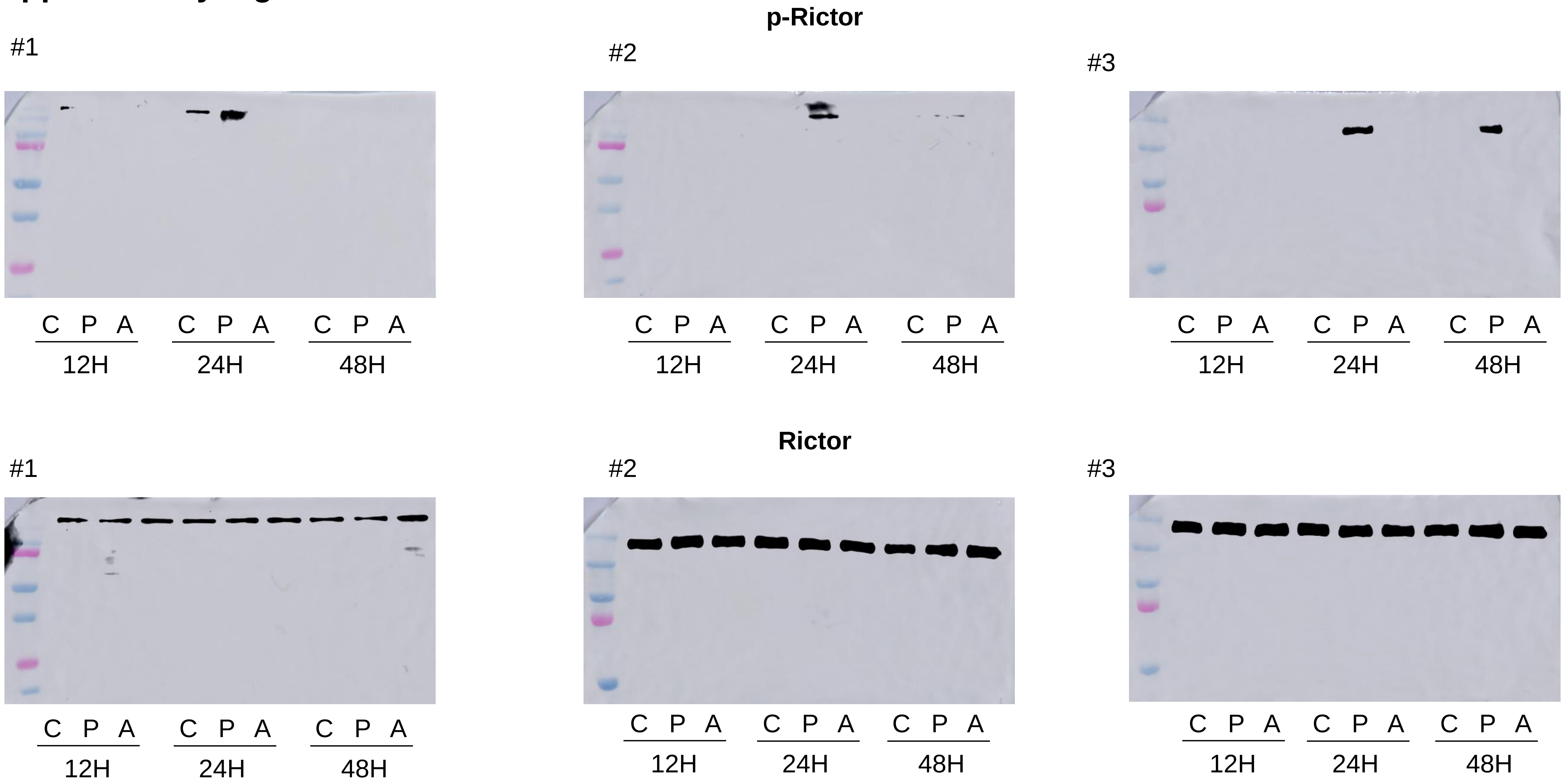

Supplementary Figure S4D

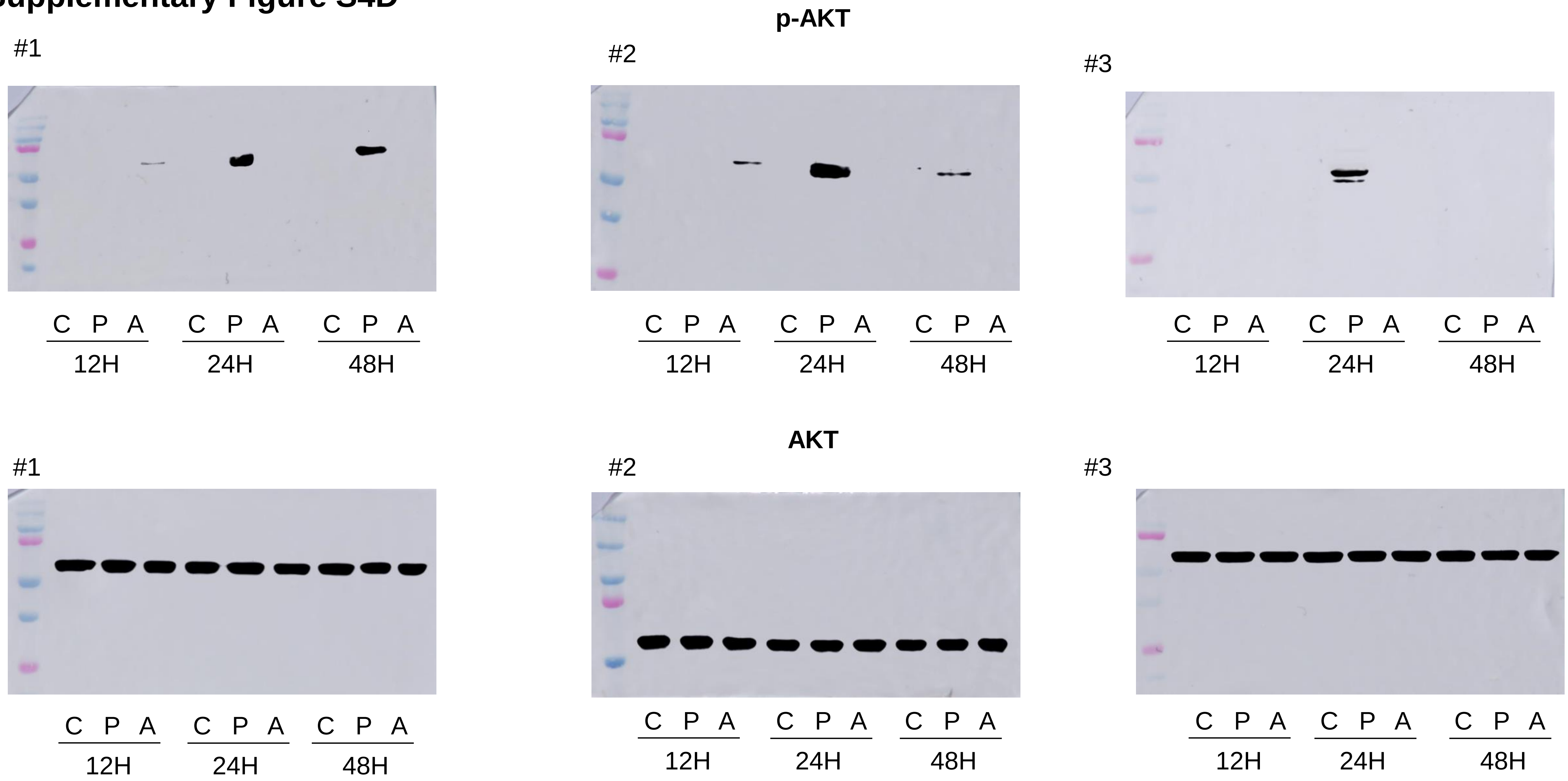

Supplementary Figure S4D

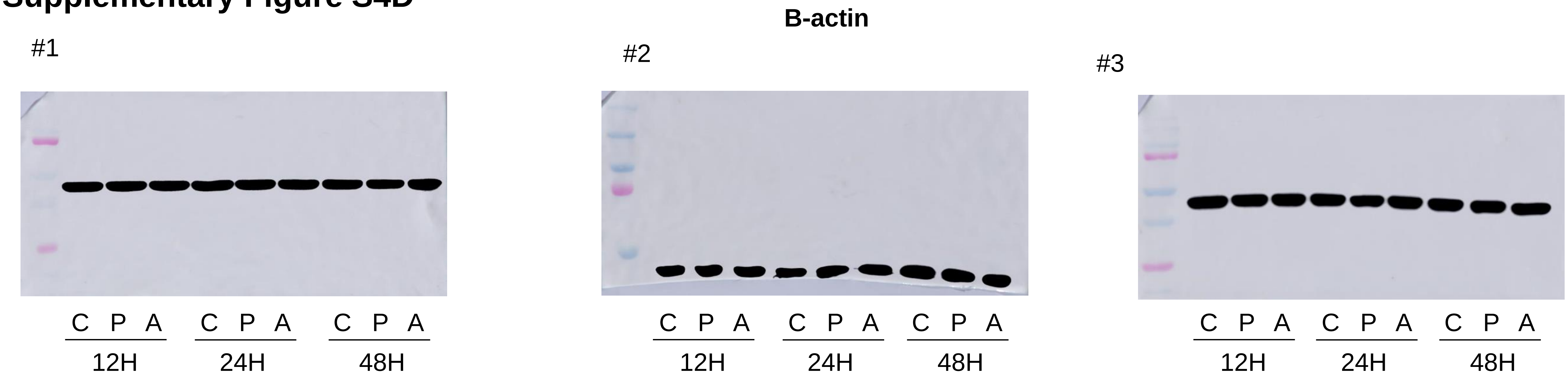

Supplement: Supplementary file 2 [file DataSheet_2.pdf]
